# Exploring medical student learning in the large group teaching environment: examining current practice to inform curricular development

**DOI:** 10.1186/s12909-016-0698-x

**Published:** 2016-07-19

**Authors:** Ciara Luscombe, Julia Montgomery

**Affiliations:** Brighton & Sussex Medical School, Brighton, East Sussex BN1 9PU UK

**Keywords:** Active learning, Medical students, Lecture, Interaction, Learning culture

## Abstract

**Background:**

Lectures continue to be an efficient and standardised way to deliver information to large groups of students. It has been well documented that students prefer interactive lectures, based on active learning principles, to didactic teaching in the large group setting. Despite this, it is often the case than many students do not engage with active learning tasks and attempts at interaction. By exploring student experiences, expectations and how they use lectures in their learning we will provide recommendations for faculty to support student learning both in the lecture theatre and during personal study time.

**Methods:**

This research employed a hermeneutic phenomenological approach. Three focus groups, consisting of 19 students in total, were used to explore the experiences of second year medical students in large group teaching sessions. Using generic thematic data analysis, these accounts have been developed into a meaningful account of experience.

**Results:**

This study found there to be a well-established learning culture amongst students and with it, expectations as to the format of teaching sessions. Furthermore, there were set perceptions about the student role within the learning environment which had many implications, including the way that innovative teaching methods were received. Student learning was perceived to take place outside the lecture theatre, with a large emphasis placed on creating resources that can be taken away to use in personal study time.

**Conclusions:**

Presented here is a constructive review of reasons for student participation, interaction and engagement in large group teaching sessions. Based on this are recommendations constructed with the view to aid educators in engaging students within this setting. Short term, educators can implement strategies that monopolise on the established learning culture of students to encourage engagement with active learning strategies. Long term, it would be beneficial for educators to consider ways to shift the current student learning culture to one that embraces an active learning curriculum.

**Electronic supplementary material:**

The online version of this article (doi:10.1186/s12909-016-0698-x) contains supplementary material, which is available to authorized users.

## Background

During the preclinical years, a large proportion of teaching is delivered in the large group teaching session in the form of didactic lectures. Whilst this is a cost effective and efficient means of information delivery, didactic teaching encourages a teacher-centred and passive learning environment [1]. Despite consistent feedback from students that lectures incorporating active learning techniques are preferable to didactic sessions, we have noted in practice, it is challenging to engage students with active learning tasks. This challenge for educators is also documented within medical education literature [2, 3].

Active learning is a student-centred learning theory which focuses the responsibility of learning on the learners [4]. This model states that in order to learn, students must do more than just listen, students must be engaged by doing things and thinking about the things that they are doing [4]. Teaching methods that are underpinned by active learning principles include the Flipped Classroom model and the use of audience response systems [5–7]. Studies comparing didactic instruction to active learning in higher education courses have found better academic outcome for students who have teaching enhanced through active learning methods [8, 9].

It is challenging to isolate the specific factors that bring about positive results in regards to student perceptions towards active learning and academic performance. Through grounding this inquiry in the context of current pedagogical practice it was possible to identify specific aspects of active learning modalities that contribute to their success and acceptability. Gaining an understanding of how students currently perceive teaching in the large group setting provides a baseline from which to form recommendations for those wishing to enhance teaching in the large group setting.

The aim of this research was to explore the mismatch between what students perceived they want and their actual preferred teaching modalities in the large group learning environment. It is unclear the way that large group teaching sessions are used as part of student learning, both within the lecture theatre and during their personal study time. By exploring student experiences, expectations and how they use lectures in their learning we hope to produce recommendations to support students in their learning both in the lecture theatre and during personal study time.

## Methods

### Theoretical framework

This enquiry aimed to explore experiences in-depth, data collected was unquantifiable and therefore qualitative methods within a interpretivist paradigm were used. A interpretivist paradigm recognises that truth is experienced differently by individuals as a subjective reality [10]. In this way the research participants are viewed as helping construct the reality of the case, with focus on the experiences and views of all the participants [11].

Data was analysed using a hermeneutic phenomenological approach with an aim of building a detailed picture of how a specific phenomenon (teaching methods within the lecture environment) is understood by those who have personal experience of it [12]. Phenomenology is concerned with the lived experience of participants. Hermeneutics adds an interpretive element, where the researcher can find meaning and assumptions within the data from participants, which the participants may have difficulty in articulating explicitly [13].

Focus groups were used to explore the views of medical students within the large group teaching environment. This is a suitable method to explore the views, experiences, beliefs and/or motivations of the students [14, 15]. Considerable value was placed upon the group dynamic, a characteristic unique to focus groups as a data collection method. The group dynamic encourages participation from those who are reluctant to be interviewed on their own and can also encourage contributions from people who feel they have nothing to say as they observe discussion generated by other group members [15]. Focus groups can empower participants and facilitate the expression of ideas and experiences that might be left underdeveloped in an interview or questionnaire. In focus groups, intra-group stimulation through dialogue with other participants is beneficial in activating memories, feelings and experiences [16].

### Setting

This study was carried out in the integrated curriculum of Brighton and Sussex Medical School, Brighton. At the time of this study, year 1 and year 2 consist of 8 modules, with patient encounters approximately every 2 weeks. Most learning was within the large group teaching environment, in the form of lectures. PowerPoint presentation slides and audio recordings are available for students to use in their own study time. Students are also taught in small group seminars, in the anatomy dissection room and other laboratory sessions. Years 3, 4 and 5 are based much more in the clinical environment, with approximately 4 days a week based at the hospital and one day of lectures.

### Ethical approval

This research was approved by the Brighton and Sussex Medical School Research Governance and Ethics Committee.

### Participants

Students in their second year of their undergraduate medical degree were approached to take part in this research. The majority of teaching to this year group is in the form of large group teaching sessions, and they had experienced this style of teaching for over one year. Key demographics were gathered from each student participant and are summarised in Table 1.

**Table 1:** Participant characteristics

Age	Number of participants
19–20	11
21–30	4
31–40	2
41–50	2
Education	Number of participants
Straight from school	8
Gap Year	5
Mature	6

### Procedure

Each focus group was 60–90 minutes in length, a total of 3 focus groups were conducted with between 6 and 7 participants in each group. The material covered in the focus groups was taken from topic guides reviewed by department of medical education faculty and piloted before use. CL moderated the focus groups and the topic guide was iteratively adjusted based on what emerged during sessions. The focus groups were audio-taped (using two devices as back up) to permit subsequent transcription and analysis [11].

### Data analysis

Data was transcribed manually verbatim by CL and analysed using thematic analysis to identify emerging themes from transcripts. CL and JM discussed the analysis, mindful of how their differing experiences and roles may influence the interpretation of the data reflecting the underlying research methodology.

For each theme that arose, the corresponding quotation was noted using the focus group (FG) number, transcript page number (pg) and student number (S), for example, FG1 Pg 23 S1. These quotations were sorted into themes and sub-themes and placed them into a preliminary table. Careful re-reading of the transcripts resulted in the final tables (for an excerpt see Table 2, full data in Additional file 1: Appendix 1) that was used to summarise findings. CL and JM met several times with members of the division of medical education to discuss the findings and minimise bias in interpreting the data.

**Table 2:** Excerpt of coding table

Theme: Reasons for participating/engagement in the lecture theatre					
Sub-theme	Codes	Sub-theme	Codes	Sub-sub-theme	Codes
Timetabling	FG1 Pg40 S4; FG1 Pg 13 S2; FG2 Pg 74 S1	Positioning in the lecture theatre	FG2 Pg 79 S1; FG2 Pg 79 S2; FG2 Pg 80 S1		
Lecturer	FG1 Pg 1 S3; FG1 Pg 3 S5; FG3 Pg 106, S2	Delivery/Questioning style	FG2 Pg 54 S4; FG3 Pg 109 S1; FG2 Pg 67 S6	Persistence	FG1 Pg 27 S6; FG2 Pg54 S3; FG3 Pg115 S1
				Consistency	FG1 Pg 6 S2; FG1 Pg 9 S3; FG2 Pg 81 S3
Perceived relevance of learning material	FG1 Pg8 S3; FG2 Pg 69; FG1 Pg 41 S3				
Student confidence	FG2 Pg 79 S1; FG1 Pg 5 S3; FG1 Pg 2 S3	Anonymity	FG1 Pg 26 S6; FG1 Pg 26 S2; FG1 Pg 27 S5		
		Motivating factors to engage	FG1 Pg12 S3		
Indirect engagement	FG1 Pg 21 S5; FG2 Pg 93 S3; FG2 Pg 93 S6				

## Results

It has been possible to construct an interpretive account of the place of large group teaching in student learning during the pre-clinical years; this has extended to the use of lectures in learning outside the lecture theatre. Several barriers stemmed from the established learning culture of the students within the institution; as such this has been elaborated on in some detail. Illustrative quotes taken from the transcripts have been used verbatim to highlight key points.

### Established learning culture

There is an established learning culture, and with it expectations on the part of students as to the format of sessions. Students were exam driven in their learning, seeing large group teaching as a means to gain factual information. The teaching session was considered as an overview of content which they would go away and learn for exams in their own time.

*“For example if they [the lecturer] said, everything on this lecture is going to be in the Knowledge Test. Every single person would pay attention to it. I mean, we’re all exam driven.”*

**Fig. 1:**
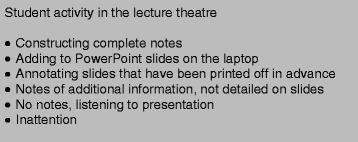
Student activity during large group teaching sessions

In the pre-clinical years, the end of module assessments are primarily based on the material delivered through the power-point slides in the large group setting. Taking this into account, PowerPoint slides were held as being central to learning. Huge emphasis was put on the quality of slides as a learning resource, for use as an aid within the lecture theatre (Fig. 1) and to have for personal study at home. Any teaching formats which deviated from providing the information needed for assessment in a slide format were not received favorably.

*“With this course the slides have to be 50 % of the whole experience. They have to be really good for the Knowledge Test and then it is just nice if the lecturers made it really interesting and clearer and thoughtful as well. That’s important too, but a definite split for me.”*

*“For me I think it is partly the lecturer, but the way our course is it is so assessment driven, it’s also the quality of the slides.”*

Students were used to a teacher centred learning environment where they were a passive participant. Although interaction and innovation in the large group setting was highly valued, students found the contrast with the didactic instruction challenging. Engaging teaching sessions were consistently stated as those where the lecturer interacted with the students, however students recognised that it was often quite challenging to initiate and maintain interaction. There seemed to be a conflict between what students perceived they wanted from the learning experience and the pervading learning culture.

*“I think most people would be engaged but not so many would participate if that makes sense. So the majority of people would probably end up doing the flipped classroom, everyone would end up listening and taking things in and that sort of thing but the amount of people that would actually want to contribute their ideas and their questions and that sort of thing probably isn’t as high as everyone who would be engaged.”*

The way that lecturers chose to interact with students heavily influenced their attitude towards interaction. If an open question was posed, it was not received very well. In contrast, it was less intimidating if options were offered to students and they had to select an appropriate answer. It would seem that a completely spontaneous and student-centred learning environment was too much of a transition and for the students.

*“He suggested answers and said, raise your hand if you agree with this, or raise your hand if you agree with that… you didn’t have to come up with the answer yourself.”*

When active learning tasks were initiated there was an element of adjusting to a new style of teaching and students understanding their ‘role’ within the lecture theatre. Students valued educators who persisted with interaction, and were consistent with the way they posed questions.

*“It can’t just be something that you do once in the lecture for half a minute and then discard because people switch on for that moment and then switch off, you need to be persistently or regularly engaging people.”*

### Intricacies of interaction

**Fig. 2:**
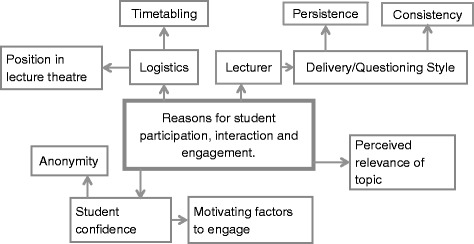
Reasons for student participation, interaction and engagement

To some extent, knowing that students’ value interaction is not enough, the exact way that educators choose to interact with their students’ needs to be considered. There are multiple factors that were stated by students as facilitating interaction in the lecture theatre (Fig. 2). Educators who came across enthusiastic about the topic they were teaching, were more engaging for the students. The delivery style of the presentation was another factor students commented on, they found educators who asked questions and interacted with the audience more engaging. The questioning style of the educator also influenced student willingness to answer questions.

There was an element of student pride within the large group teaching environment, students stated that they often didn’t have the confidence to speak up in-front of their cohort for fear of getting the answer wrong. Those teaching sessions where students were given the opportunity to answer as a cohort through ‘hand-raising’ were perceived as less intimidating. In the same way the use of audience response systems allowed for all students to consider and answer questions, in an anonymous way.

*“What stops you from interacting”, ”Feeling stupid. Probably saying something that looks really stupid.”*

*“I think you get more participation with the app. It’s anonymous. But at the same time you are still finding out the right answer and thinking it through.”*

Sometimes the amount that students engaged with the teaching session was dependent on how relevant they perceived the topic to be. Educators illustrating concepts with clinical anecdotes and cases helped engage students.

*“ I think it is about linking it, to what is relevant in the future”*

### How lectures are used in learning

The way that students use lectures in their learning related to their expectation of the lecture experience. The majority of ‘learning’ was perceived by many students to take place at home.

*“For me, most of my learning occurs at my desk, at home.”*

**Fig. 3:**
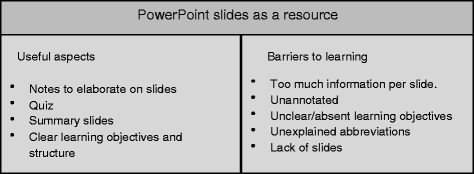
PowerPoint slides as a resource

Taking this into account there were many resource features that students found beneficial to facilitate revising at home, and others which acted as barriers to their revision, as summarised in Fig. 3. Students found slides that had a clear structure, with learning objectives and a summary useful for revision. PowerPoint presentations where lecturers had elaborated on the slide content in the ‘notes’ section were regarded as beneficial. Where educators had included a quiz students found this useful to test their knowledge, it was also used to highlight the key learning points of the lecture by the students.

As slides were so central to learning, the absence of slides was very frustrating for students as they had no resource to revise from. Slides with poor structure, lack of learning objectives and the use of unexplained abbreviations were all perceived as unaccommodating when trying to revise. Slides with large volumes of information were challenging to use as students were unable to identify the key learning points they were supposed to take away. On the other hand, slides with little, or no information, which were mainly pictures were also unhelpful when trying to learn from the slides in personal study time.

## Discussion

The results from this research suggest that there is an established learning culture and with it, expectations amongst the students. This has multiple implications with regards to implementing an active learning curriculum.

The design of active learning tasks, such as those within the flipped classroom model are often based on andragogical adult learning principles [17]. Andragogy is the method and practice of teaching adult learners and is underpinned by 5 main principle assumptions set out by Knowles [18, 19]. These principles lead to underlying assumptions on the part of the educators; students will embrace an active learning curriculum and students have the characteristics of the adult learner. As the students involved in this research were in their second year of university, an underlying assumption was that they would have the characteristics of an adult learner.

Some of the experiences shared in this study illustrate andragogical principles [19]. For example, students stated they were more likely to participate in and engage with teaching sessions if they perceived the material to be relevant. However, in some cases, the student voice suggested that they had not developed the academic maturity to function as adult learners, especially with regards to their motivation to learn. A major factor for student learning was the external motivating factor of assessment. The students appeared exam driven in their learning and were concerned with what they needed to know for the knowledge test.

Motivation is one of the three non-cognitive processes which have been shown to affect adult learning [20]. Motivation for adult learners is based on internal factors, such as the internal desire to succeed and presence of personal goals. With medical students it is hard to distinguish between what can be considered an external motivating factor and what is internally motivating for the student [21]. Currently, the way medical students are selected, taught and examined, conditions them to strive to do well in examinations and encourages an exam-driven culture of learning [21, 22]. For many, the internal need to succeed may be equivalent to passing exams to a high level. By acknowledging that not all students conform to the andragogical principles, educators can adapt teaching methods to suit the established learning culture of the students.

Understanding what motivates students can enable educators to tailor teaching to appeal to how students function as learners. Examining the approach that students take to learning can also be beneficial in designing and delivering teaching sessions for students. Within higher education it is recognised that students take different approaches to learning; deep, strategic or surface approaches [23, 24]. Through identifying the approach students take to their learning, one can consider the implications for understanding and information retention. A study carried out with a cohort of medical students found that those who took a deep or strategic approach to learning performed better academically in final year examinations compared to their peers who adopted a ‘surface’ approach to learning [23].

Whilst the student approach to learning was not the sole focus of this enquiry, the data collected suggests that many students adopted a strategic approach to learning within the large group teaching environment drawing on a combination of deep and surface learning approaches. Motivating factors for learning and participation in the sessions revolved around gaining enough information to pass the knowledge test, with some thought for vocational relevance later in their careers. Whilst this can be effective in passing the knowledge test it can result in patchy and variable understanding [23]. Through understanding the student approach, educators can monopolise on this and tailor sessions based on factors which motivate students to learn and engage.

## Recommendations

Taking into the account the established learning culture of the students and perceptions towards innovative active learning methods, the following recommendations have been formulated to aid those who are involved in large group teaching.

1. **Use of interaction to engage your learners.** Interaction is a powerful and simple tool which has the capacity to engage all students in the teaching session. By posing questions to students they are no longer passive participants in the lecture theatre, but use higher cognitive thinking skills to consider and answer questions. Although it may not be the case that every student answers a question, the process of thinking through and comparing their own answer to that of their peers acts as a form of indirect engagement.
2. **Persevere with innovative active learning methods and interaction.** It is not uncommon for students to need time to adjust to a new method of teaching, with positive perceptions towards the flipped classroom model developing over the course of a module [25, 26]. At one institution structured interactive sessions, were introduced for students to enhance interaction in the lecture setting [27]. Through planning and organised efforts on the part of educators, there was a significant increase in interactions per student over the course of the module.
3. **Use of digital technologies to structure interaction in the large group setting.** The presence of structured and planned attempts of interaction within a session has shown to increase the amount of interaction per student over time [28]. Audience response systems [29] provide a simple platform to engage learners and initiate interaction. These systems have the additional benefits of anonymity for student learners, and the opportunity for educators to check student understanding of content covered within a session.
4. **Understand the students' approach to learning within your institution.** Alternative teaching methods underpinned by student-centred learning theories make assumptions with regards to student motivations for learning [17]. Some students may not have achieved the maturity to function fully as adult learners, driven by internal motivating factors [17, 18, 21]. Where this is the case, it may be appropriate to introduce external motivating factors to engage learners. For example, where low stakes assignments have been introduced to the flipped classroom learning model, learning outcomes have improved [27, 30].
5. **Be aware of student expectations of the learning experience.** Students may have set expectations as to the format of large group teaching sessions based on their previous experiences. If this is the case, they may be uncertain of the role required of them in the learning environment when new teaching methods are implemented, and become disengaged with the session. In a flipped classroom course redesign for pharmacology students, educators emphasised their expectations that students would review offloaded material before each class and actively engage in in-class activities; this was thought to aid the transition for students to an alternative teaching format [6]. Although prior to the course students stated a preference for traditional didactic methods, after completing the course, the flipped classroom model was the preferred method of instruction. Signposting clearly articulated expectations of learners in student-centred environment has the potential to empower students in their learning.
6. **Use of teaching strategies that will appeal to the established learning culture of the students.** In the context of this study, students were assessment driven in their learning. Educators can monopolise on this learner characteristic to engage students in the session. Digital tools such as audience response systems can be used as a platform to allow all to students to anonymously answer questions posed by the educator. Using quizzes and tasks that mimic the assessments students are working towards may engage them in the teaching session. Another strategy is the use of case studies and clinical anecdotes to engage students. The extent which students interact in sessions is influenced by how relevant they perceive the content to be. Illustrating key learning points will aid students in understanding how the content will be clinically relevant to them in their future careers as clinicians.
7. **Be transparent and communicate clearly with learners.** For some students they did not appreciate the value of alternative teaching methods in enhancing their learning. Models such as the flipped classroom were dismissed as being too much additional work, without thought to the active learning they could facilitate. Explaining motivations for changing teaching practice and the pedagogic theory supporting their implementation may inform students as to how they may enhance their learning experience [6].
8. **Embrace the multiple roles of the medical educator.** Students view lectures as a method of imparting factual information, where the content could be revisited outside the lecture theatre in their wider learning and revision. The role of the medical educator as a resource provider was emphasised alongside their roles as a teacher. Where in class exercises are passive and not consistently well designed, students will be reluctant to engage with them [17]. Consider the learning of your students holistically, learning takes place both within and outside the lecture theatre – ensure that their learning is supported in all environments.

### Limitations

It is important to note that there are some limitations to this study.

Convenience sampling was used for student participants; those who volunteered may have done so because they felt particularly strongly about the way large group teaching sessions. In addition to this, the sample number of participant (*n* = 19) may not have been truly representative of the cohort as a whole if the student sample was particularly supportive of lectures or particularly against lectures as a teaching method.

Focus groups as data collection method have their own limitations, the group dynamic and pressure can cause possible exaggeration [16]. At times the students talked over each other and certain members of the group dominated the discussion; this can be unhelpful when the dominant voice is not challenged by the group and solely their viewpoint emerges from the discussion [31].

The students who took part in this study were in the second year at a UK medical school which follows a spiral curriculum. As such, whilst these findings and recommendations may be transferable to other similar teaching settings, they are not generalisable.

## Conclusions

Presented here is an account of student learning in the large group teaching setting. Through examining the experiences of students in the context of existing pedagogical theory and research it has been possible to provide a series of recommendations to aid educators in engaging and supporting students in the large group teaching setting.

This exploratory study consisted of mature students and students straight from school. In light of educational theories around adult learning it would be interesting explore in a future study any possible differences in approaches to learning between these two groups of students.

The established learning culture of the students has extensive impacts on their perceptions and approach to learning. Short term, educators can implement strategies that monopolise on the established learning culture of students, to encourage engagement with active learning strategies. Long term, it would be beneficial for educators to consider ways to shift the current student learning culture to one that embraces a student-centred, active learning curriculum. It is likely that this study has transferable findings applicable to many healthcare education settings.

## Abbreviations

CL, Ciara Luscombe; FG, focus groups; JM, Julia Montgomery; pg, transcript page number; S, student number.

## Additional files

Additional file 1: Appendix 1. (XLSX 15 kb) Additional file 2: Appendix 2. (DOCX 97 kb)
