# Supplementary material for: Exploring medical student learning in the large group teaching environment: examining current practice to inform curricular development
Source: BMC Med Educ. 2016 Jul 19;16:184. doi: 10.1186/s12909-016-0698-x (PMC4950633; doi:10.1186/s12909-016-0698-x)
Supplement: Additional file 2: — Appendix 2. (DOCX 97 kb) [file 12909_2016_698_MOESM2_ESM.docx]

Transcripts

# Focus Group 1

I: So first of all, I just want you guys to tell me about a couple of lectures, within module 203, that you have enjoyed, or you’ve really learnt a lot from and engaged with.

S1: There was this, erm, I don’t know who it was, the Australian? (agreement from group) and he was very engaging. So he had, a case up on the slide and he’d ask questions about it. ‘Hands up who would deal with it this way, hands up who deal with it this way’. And stuff like that..

AS: (agreement from all members of group)

S1: I came out of that feeling like I had learnt quite a bit.

S2: Was that the ovarian cysts one?

S3: he was very encouraging to make us participate. He did it in a very nice polite way, I thought, which makes a big difference. We’ve had some lecturers who are sort of a bit arsey. (laughter) They’re like ‘Ohh nobody is answering these questions’, sort of thing, or if you get it wrong … So he was quite encouraging. And he did get a lot of audience participation. Which was good. (agreement)

S1: Eventually, Yeah.

S6: It’s better I think when they do it as a hands up because then no one feels, well I don’t feel, picked on.

S1: Exactly.

S6: The pressure to be like om my god I have got to get this right, sort of thing, so it’s a lot easier when, it’s everyone and you just sort of put your hand up in a way. It’s still thinking about what you are trying to do.

S2: cos we had that in the diabetes symposium,

S4: Yeah

S2: Yeah, pick True and false. Sit down if you do this, sit down if you do this. Until there was no one left.

S3: It had quite a good impact because it was saying, actually we don’t do any of the things, sort of demonstrating within the whole year,

S2: how we’re all really unhealthy.

S3: How difficult it is. doing a physical demonstration, makes a big difference, rather than sort of, saying, ah 2% of people do this, they could just say a stat on the board, but showing actually within our year, we think we’re all healthy people, to, what’s the word? Visually see.

S2: And it was our second symposium of the day. (Agreement)

S3: I think with that one as well like, when we have questions, when we have lecturers ask us questions, there tends to be one or two students that will only answer them. The only people in the year who will answer the questions. And they are only answering them, not because they want to show off, a lot of the time, because nobody else will answer questions. We’re quite a quiet year in terms of not being bold to answer questions a lot of the time, erm, but with the Australian guy, because we were, he relaxed us all and we felt comfortable, when he was asking questions people were answering, and different people were answering.

S2: Did we have him for two hours in a row?

S6: I think it was a two hour session, yeah.

S1: Yeah.

S5: But also because he suggested answers and said, like, raise your hand if you agree with this, or raise your hand if you agree with that. It’s like you didn’t have to come up with the answer yourself, you could just say, you know, what you thought,

S*: You could think logically about why you would do it, instead of trying to ….

S5: Exactly, think what you were doing to put

S*: …it puts pressure off you

S4: I think some lecturers sort of ask you a very open question, like, without giving you any options, and if you have no clue, at all, you don’t even know what type of answer they’re looking for, it can be really difficult but when, they do the whole, say if you think it is this or this, it just gives you a lot more confidence, to participate I think, cos you actually aren’t going to say something completely ridiculous, which everyone is going to laugh at.

S:6 I find when they keep using that throughout the whole lecture as well, because you can keep building on what knowledge you already have, were as sometimes they’ll sort of do it and go off on a tangent a bit, and like,

You feel like you’ve gained in that lecture as well, you can see the knowledge you’ve gained. Cos he was asking the same sort of question, wasn’t he?

What would you do for each case?

S3: And the first one, we basically, we all got it wrong, and then he explained why, and by the end of it we were getting it right, so you could see, that you’ve learnt. And you come out of it feeling more satisfied that you would otherwise,

S6: Yeah definitely.

S5: yeah, he gave us different options of treatment, for different cases, surgery, vs more conservative, versus observation or something. And asking us the questions throughout the lecture for different cases, and we got better at it.

S6: But at the same time the information was on the lecture slide, so I’m not like erm worried about writing down all this information that has been said, even though it’s like recorded, it’s still, difficult to then come back and be like which part.

S4: I quite like it when they put a little test, a little quiz at the end of the lectures as well, it’s sometimes hard because sometimes people sometimes get up to leave, and the lecturer is there like trying to talk but it is helpful for I think, as you say just summarising at the end, if you, if you’ve like been paying attention in that lecture, and you can actually like answer things which you wouldn’t have before, and it’s quite good when you come back to revision, as well I think to look at the little quiz.

(agreement)

S1: Definatley

S5: And who told us last year its better to test yourself before revising something again,

S2: I don’t like the little quizzes at the end

S4: Do you not?

S3: I don’t think I like them because I don’t know the answers.

S1: Why is that?

S2: That’s why I don’t like the, because we just get the questions, and not like, or they, erm, or they go through them in like 30 seconds at the end, so you can’t write down what the answers were.

S3: I think with on that, having another slide afterwards or the ideal answer,

S6: having the notes bit underneath.

S4: yeah

S3: with the erm, questions, erm, in terms of people walking out of the lecture, what some lecturers do better than others, in terms of rather than saying okay that’s the end of the lecture now we’ve got some quizzes. When someone hears it’s the end of the lecture half of our year, walks out.

S1: switches off.

S4: as soon as they say…

S3: If they were to say, have the last slide, but not do it in a closing up way. Just have the last slide and say, go onto the next slide with questions and it to be a continuation of the lecture, rather than ‘the end of lecture, now questions’. People have engaged before much more with that. And also with the confidence in the year, rather than, asking the group, asking the year, Oh what’s the answer, saying, who thinks it is ‘A’, everyone raises their hands, who thinks it is ‘B’, everyone raises their hands, because people are much more confident doing that. (Agreement)

S2: We’re better at answering in a group.

I: You said with the Australian lecturer, you had people joined in ‘eventually’? What eased them into that?

S2: Because he did it in the same way each time, after the first one we figured out what was going on.

S4: you figured out what was going on.

S2: like what he wanted us to do. And he was quite funny.

S5: also, he was quite blunt, he was quite funny and quite blunt, you know, when people didn’t answer he was like, ‘come on everyone get involved, everyone do it, you’re not going to get penalised for any wrong answers. (agreement)

S1: Yes, that’s what I thought helped, he would then pick on people, and was like, okay why did you say that, and a couple of people would tell him why, and he wouldn’t, erm, make fun of you for not, knowing the answer, and it was really important for getting people engaged.

S3: He wasn’t patronising, he was nice, he was friendly, we’ve had other lecturers who have been quite, patronising, and a lot of people, switched off completely, and I just got on with essays, when he was sort of really rude and like, and not engaged with that. I think with this guy he built rapport, and then through the mechanisms people have said.

S1: yeah definatley.

I: so S4, you touched a little bit on, you said like erm, only some students answer questions consistently, in other lectures. So what stops, all of you guys from erm, answering a question, you said it’s, because questions are a bit vauge.

S5: I think its because people find it a bit intimidating, because everyone is listening to you, and most of the time you are not sure, because it is not something you have covered before, so you don’t want to get it wrong in front of everyone else, and so those,

S4: embarrassment

S5: ..what made us join in to the other one was because he’d offered the answer already, so you knew it was one of those answers so it’s less chance, that you are wrong, And also…

S1: and other people had got it wrong

S5: also, yeah other people had got it wrong. And he was really encouraging, as well, I think that helped.

S4: What I really liked about him was that he, so, you knew, he wasn’t going to make fun of you if you got it wrong, say. With a few other lecturers we’ve had I think, recently we had someone say, ‘Oh who’s the smartest person in this year, they can answer this. Which, actually you know, it is actualy that, for a start it puts a lot of pressure on that person, and it doesn’t give you much confidence to answer later on I think.

S2: It wasn’t a very fair question to ask.

S4: No, And I dunno you sort of, I like erm, Julia Montgomery is quite all for just us learning, things that we are actually going to need for future practise and like, tips and what she sees like day-to-day. Where as...

S2: I’m trying to think of who the last imaging guy was.

S4: yeah, that was him. One of these imaging lectures we just had, it was all very sciencey, and like, ooo who knows what this is, who knows what this is, very sciency and not like, encouraging I think.

S6: I feel like he was sort of pitting us against each other,

S4: Yeah, as if whos the clever ones here? Which I don’t know, I didn’t feel like that wasn’t a very good atmosphere to bulid in a lecture.

S1: That’s like a complete contrast to this austalian.

S4: to the Australian guy, yeah. Who just encouraged us.

I: you were saying that the Australian guys lecture was quite case based, (agreement) Do you think how much you engage is sometimes dependant on the subject matter (agreement) cos you’re all obviously thinking about becoming doctors.

S2: it didn’t have to be cased based, he could have done it without using cases, but, it worked.

S3: I think it is about linking it, to like, what is relevant in the future, so we’re learning about, the attachment of this muscle and the attachment of that muscle, I can’t… enage.

S4: I switch off in that.

S3: What relevance is this going to have? When I’m… I dunno… In future medicine whatever you go into like, I’m not really going to, like, need to recall, this muscle attaches to that muscle. It’s whether it’s clinically relevant, so when you have a case, you can see directly how it is clinically relevant. Where as other things, maybe for them, for us to engage, for them to explain why, it’’s clinically relevant or important, in the future rather than just being, some sort of science, I’m learning now but don’t need to know in the future.

S5: I think also, case based information is, more, there is a logical progression to it, so you can sort of, you can explain how you, got to answer and how, you deduced that. While if you, if it’s pure scientific questions, it’s just a fact, and you have to be able to know the fact, and work it out from that.

S4: I think the good thing about the case based as well is that you are not always going to treat every patient with the same illness in the same way. And I think when you have just have a list of say, Asthma, these are the different meds, it doesn’t really give the broader picture. Where as if you had, like, three cases. This lady came in with this and normally we would do this but because of this we did something different, it just makes it a bit more, erm, less just list learning and more, actually think logically about, patients and I think everyone, at the end of the day that’s what we want to do. So I think everyone engages with that quite well.

S2: Like when they give you a case in the right order, so, where people just give you like, this is the disease, these are the symptoms it presents with, this is what you do, its not the order you’re ever going to see it in. You see the symptoms first, and then everything else…

S3: Things click quite well as well. Was it Julia Montgomery’s, I think it was the MT group she did. Where you were presented with a female, no periods, for this long, and this is when her last thing was. She had a case and then, she went through the case, she explained the case, and explained how she got to the answer of what was wrong with her. Going through different possibilities, explaining why they were wrong, and why the right one was right, but not just one case, doing another one. So the first time, we were all baffled, like, what is going on, the next one was like oh, that makes sense because that makes sense, or, she’d had that before. So by the last sort of one, however many she did, things fit, you understand, you understand things, because you can link it and you can see where things are coming from. That’s a really good way of doing it.

I: So how feasible do you think that kind of case based, putting it in context is, with the volume of information you guys are expected to know for your KTs and things.

S4: I think it is good to almost, some lectures we’ve had, the first half has been, the theory and the second half has been like clinical application, and I think that works really well, when you learn sort of facts and things behind it, then afterwards you just have a few, cases, um, and they’ll be like a clinician saying, oh in practise this is, what we see and applys it, that would be quite feasible with a lot of the lectures we have.

S5: I agree that’s helpful, to have it structured that way. But I disagree in that I don’t think many of our lecturers do that now. I think most of them are mostly theory, there are only a few of them that have included clinical cases and things like that.

S3: I think with, you know the whole, testing your knowledge is better than re-revising it. Starting off with a case, saying this is the case. Questioning like, cos we are naturally scientists, scientific people and actually questioning, well why does that do that. Why could that be, why could that be. And then doing the theory behind it, and then redoing the case at the end, so in the theory, we’ve been thinking about the case and then, learning the knowledge to work out the case. Sort of works.

S4: I don’t know, sometimes I find with that though, if someone just gives like a case we haven’t learnt about at the start of the lecture, I sort of get ah well how are we supposed to know, I have no clue. Sometimes that just makes me switch off. But it depends how it is worded.

S3: If they say like what, I’m not expecting you to know this, explaining the case, from this lecture I hope you to know this by the end,

S4: yeah, explaining, yeah, yeah that would be good.

S6: I don’t think it was this term but in other terms, they’ve done part cases, so they’ve put like the starting scenario and then go through the differential, then come back to it and think, okay so, they’ve got this symptom as well, so what does this mean. I found that quite important, putting it onto a person, instead of, just like listing, I just can’t do that. It just doesn’t work so.

S1: I guess the problem with the case things is its not really, we’ve got to learn physiology and pathology but we can’t really apply that to much, well I guess you can for pathology, but for physiology and the actual basic sciences, it’s actually really difficult to add cases, that’s the other aspect to it.

I: How do you find the physiology lectures and things?

S1: Erm who have we had for physiology? I always think in terms of which lecturer I have which probably suggests something, erm who have we had?

S*: Paul gard

S4: Paul Gard has done a lot of the endocrine, it depends on the lecturer.

It depends how it’s split up its usually Harry Witchell in every module.

I don’t think it’s the physiology that is boring I think it’s the lecturer.

S4: Yeah – it depends so much on the lecturer, even some of the ethics lectures we’ve had. Which I think is quite an interesting topic, I haven’t found that engaging, because I have found the way it is presented ..mm.. not very engaging and not, just more of like a discussion with the lecturer talking at you. Whereas I would normally find the ethics probably quite interesting.

S3: I think ethics is more interesting in a smaller group though. Because then everyone has a point to say and every one can discuss it. And that might be something that, because we have only had one or two…

S*: I think it was 3 (agreement)

S3: You could almost have in a small group of you, maybe 10, quite nice.

I: Have you ever had lecturers say to you we’re going to get into small groups within the lectur theatre, no.

S4: No, we’ve had discuss with the person next to you.

S2: But I dunno that doesn’t really work. I don’t think, because people don’t discuss, what they are supposed to be discussing. A lot of people will just have a chat.

I: Why is that? Why do you think people chat about unrelated things?

S3: because they are lazy. (laughter and agreement)

Uninterested.

S4: I think also, if you are not sitting next to people, next to a friend, people find it awkward so they just stop trying.

S1: Or they give us too long maybe

Yeah

S4: there is too long sometimes and you’re sitting there like..

S1: You’ve got 5 minutes to think of three things, and you’ve thought about them, then that’s it, I don’t know any more, maybe that its.

S3: Maybe because there is no … consequence, of not, doing it. If we have to discuss it right here, right now, then you are observing us. We’re not going to start talking about last night, because you can hear us, where as, in the lecture hall there is no kind of consequence, they’re never going to know whether you are talking about last night or, talking about the ethics. Unless they have some sort of way of erm testing that you did talk about it afterwards. Saying oh I am going to pick on people to answer the question, or tell me their view or something. So some way of threatening is the wrong word, but blackmailing people, that’s the wrong word as well. People into like actually discussing it. Some sort of authority on the matter. (18.56).

S4: That’s why MT groups work quite often I think, because it is too small a group to be … you can’t really, go on your phone, or talk to the people next to you, or like write an essay, or any of that, where as in lectures, you so easily can if you wanted to,

S3: a hell of a lot of other There are a lot of people who are either asleep, or writing essays or revising something else, or what you.

S6: I think they are too long though as well, not matter how focused I am, about 35 minutes in and I’m done.

S2: I can do a whole lecture, it’s just 7 in one day.

S6: I can’t unless they’re very engaging,

S3: Like half way through, it does work if they make everyone stand up. And sort of jiggle about.

S6: If they’re engaging and asking different things from you then, I can stay focused. But if it’s just them giving the lecture and trying to stay focused and take notes, I just switch off after 35 minutes.

I: How useful do you find it taking notes in lectures?

S3: It keeps me engaged. I fall asleep otherwise. (Agreement)

S2: I was falling asleep this morning when they turned the lights off. That was unhelpful (agreement).

S3: that’s definatley something.

S2: We had a whole lecture in the dark this morning, it’s not ideal.

S5: You just had this… but no one said anything.

S1: I think with notes I personally don’t write, the things down that are already on the slides, just extra stuff and maybe a bit of understanding. So I can go through it later, (agreement) I don’t know, for me, most of my learning occurs at my desk, at home, (agreement)

I: Would you all agree with that?

S1: That’s for me anyway.

S3: It depends whether I listen or not. I find, erm, I get a lot more out of the lecture if I listen, which is a bit obvious, but… It depends on my attitude when I walk into the lecture, so it’s a lot of things the lecturer can’t actually change.

S2: I feel like I need the lecture for things to make sense, I won’t remember it but having had the explanation, means I’ll understand it when I go through the it later, (agreement – yeah exactly) where as if I just had the slides and just ahd to make notes on it. It would make, and lot less sense.

S4: It would be so much harder, and also, I think, I know for me, I relate information, I picture, the lecturer or sometimes I like, relate it to something, like a specific thing they said or like the Sex Therapist who came in and made us all stand up and do funny things. You’re not going to forget that, like I dunno, you just link things, just things like that.

S3: Cos some people are quite visual learners I’ve been told I’m a very visual learner, so drawing diagrams and things like that, cos sometimes they do draw pictures on the paper board, it does help, when they’re showing where things come from,

If you can see it (agreement)

S3: … If you’ve got good enough eyesight.

S2: It doesn’t help when the lecture is just pictures, and they haven’t included any words,

S3: Yeah, when there is not enough information on the slides,

I: Is that for your own learning when you get home? It’s not useful?

S2: Yeah, because you go back and its just …

S3: There’s no information there.

S2: You don’t know what their point was. It’s just a picture of something.

S4: If there is Camtasia though, then that’s very useful.

S*: If it works

S2: I don’t ever use Camtasia,

S4: I don’t very often, only if

S2: unless I didn’t go to the lecture.

S3: I think the worst thing ever in a lecture is just to read mainly off the slides, they don’t tend to be lecturers form BSMS, like, hospital doctors, that come in

People that only come in for one lecture

Well yeah and they literally, when they’re just reading off the slide, not. Cos it makes no difference me listening or, not because its just, I can do that at home I can just see what it says, but when there is explanation or understanding or things like that. Then it makes it worth listening.

S4: I think the worst lectures are those when I, cos I’m the same as X, I just write down extra bits, it’s just when, you’ve literally written two sentences, because everything else has been on the slide and you sit there thinking, I could have read that, but, yeah.

S5: for me, lectures are so that I don’t go into that lecture blind at home, so that I sort of have, an understanding and I have some knowledge of what was going on, rather than just opening an completely new topic.

S6: For me, the lectures are literally just so I don’t miss any information, that they have said and not written down. Like, I actually do better when, I listen to it through my own recorder, then cos, If I get lost I can look it up, because I don’t feel able to put my hand up and say go back I don’t understand what you have just said. Whereas when at least I’m at home I have time to pause it, go and find it out or go and ask someone, and then have that understanding which then I need to continue the rest of the lecture. I mean if I miss a point, early on in the lecture, there is no point, I won’t understand it, but I will try and get as many notes down as I can.

S4: To go and help you when you get home …

S1: I dunno yeah, for me I think, that lectures, I think should, the best ones that I’ve found, are the ones that focus on the understanding first, and not necessarily go through what is on the slides, erm, cos you can do that at home, but as long as you’ve got the understanding there, it will definitely help when you, and it will help, your understanding. (Agreement) You know the sooner I go through a lecture after I’ve had, been taught about it sticks in my mind more.

S4: A lecture, which I thought was really good, which is quite unusual was from Julia Montgomery, she gave out like two pieces of paper, a diagram of the ovarian and menstrual cycle, and she didn’t actually go through the slides of the lecture, very many of them, we just spent most of the lecture, going through that diagram.

S3: Yeah that was really good.

S2: I didn’t like that.

S4: I found that really useful cos then, the slides were just really, quite simple afterwards, but her explanation of it, I thought really made a lot of sense.

S2: Cos I felt like that was something that had already been explained to us, so I didn’t feel like I needed to go over it again.

S4: It had at school but I don’t think it had at med school for me, at secondary school.

S3: No, I didn’t know it either and I thought it was really good.

S2: Are we talking about the same lecture?

S4: I’m talking about,

S2: I’m talking about the one with Anna Crown and Julia Montgomery where they did half each.

S4: Yeah, yeah.

S3: No, wasn’t there a different one, where she handed the forms out, the sheets out, with just her.

S2: This is the one where they spent the first half, the one I am talking about they spent the first half, drawing and skipped half the slides to get to the end bit, and I didn’t like that. Because they lecture slides are the information they ask us about, and I feel like,

S5: I don’t mind when people try alternative teaching methods but then, if they forget the lecture slides in the end I feel like, sometime, what the, you know what else they did is sort of in addition to the lecture slides so there is more to learn in the end. That was well as what they are doing so, I dunno.

S4: I think the lecture slides most of them were sort of like clinical, I can’t even remember what it was about, it was about turners syndrome and things, so it was very clinical stuff. But we spent the first part of it, cos you had to know about the menstrual stuff in order to understand it. I thought that was done a lot better on paper and diagram than like a slide of words like, the menstrual cycle blah, blah, blah. I don’t know, I just thought that was good.

S3: I liked having a physical piece of paper in front of me, cos I never print anything off, I do it all on the computer I liked having a visible piece of paper in front of me, with the diagram which I could then annotate it with what they were saying. And I could see it in front of me and I could follow, with a pen. I’m quite sort of visual in that respect.

S2: it’s more the skimming through stuff that I object to. So they produce the slides and then, don’t use them. Or they say, don’t worry about this, you can do this on your, own, or this isn’t important. I feel like if they’re gunna write the information down and then put it up there they should go through it.

S3: I assume with that though that it’s a simple sentence like the cat sat on the mat are the slides that they’re skipping through where, the cat did just sit on the mat and there is no point me saying the cat sat on the mat. There is no point me saying it I might as well say the elephant had pink ears because he did. It depends what they are skipping through….

S4: yeah it depends what they are skipping through.

S3: if it’s important information that needs understanding that they should verbally explain to us or whether its just about facts where there is no point in them wasting time.

S5: I liked, erm, Paul Gards thing with the menstrual cycle, I don’t know, I found that lecture, really helpful, it was literally, he just had a really big diagram, with everything that happened, and he split each part up and went through each bit. So like hormones, the endometrium, the whatever else there was, the follicle, and he split each part up and went from the beginning to the end and explained every single one. All we had was the diagram but he explained it so well and at a reasonable rate so that we could take notes quite easily, and I found that really easy to understand. I think the amount I learnt form that lecture was a lot.

S2: I think it would have been nice if he had given us some notes, cos that lecture was literally just a picture, wasn’t it. So if you missed anything, then you wouldn’t have had it.

S6: true, but I found that quite engageing.

S4: Yeah but I think that forces you to listen in a way. Cos I found like everything he was saying was quite important. So I was actually listening in that lecture quite a lot.

S6: Although. I feel sometimes, if you are so busy taking notes, then how do you actually process what they are saying. Its all just a, I can leave lectures making loads of notes and I don’t actually know what they’re talking about sort of thing.

S4: That’s why they have Camtasia so that you can play it back.

S6: That’s why I record it myself and I go back make notes.

S3: If it works though. I think there is an element of the way someone speaks, that can make a big difference to it, to learning or engaging. If someone speaks too slowly, I find that I really hate it, and I get bored and like, just get on with the sentence. Or pauses half way through the sentence,

S2: I find it really patronising.

S6: Or the other side, you have Anna crown who is like *click click gesture*, who is just to fast

S2: 100 slides in an hour,

S3: Yeah, you’ve lost it, you’ve lost the plot, you don’t listen to anything and there is no point like, you can’t engage because, you’ve lost where it is, I think, in between pace where they are talking at a reasonable level where you can, stay engaged whilst taking notes, erm so sort of a half way in between. Definitely like pausing half way through a sentence, saying erm every 5 seconds, is very difficult to stay with that. And not think, oh in this time I could have replied to a message or something in the time before where they actually finish a sentence.

I: Fair enough

S5: And also for going back to the asking questions, I’ve heard a lot of different opinions on when lecturers ask questions how many, how much time they should leave because, some people have said, I’ve heard other people say a lot of the lecturers leave too much time so that you know, they just get too bored and they switch off, but then other people for me personally I actually quite liked, not it the, I remember we talked about the small group where you start talking other things where… or when they ask quick questions in lecture when they leave lots of time waiting for people to volunteer answers, I find that useful because it give you time to actually think about it. When someone answers straight away, which is usually one of those few people who raises their hand. Then I don’t actually think about it because I think, Oh wait, they’re just going to answer it. If they just leave a bit of time for everyone to think about it before selecting someone, I find that useful.

S2: I think even if I know the answer, I wouldn’t volunteer it, even if I was certain.

S3: That’s two separate things though isn’t it.

S1: I think that is the same for me as well.

S4: I don’t like it when people shout out as well. I much prefer it when, people put their hands up for that exact reason. Cos I think, you know that someone is going to shout out the answer, whereas, if its if like a hands up thing…

I: So you’ve talked a lot about slides, you’ve also, had use of the flipchart perhaps? How do you find that?

S3: It depends what they write on it. If it’s something that useful, like to visually see and they put it out, there have been some diagrams that have been absolutely pointless. If it worthwhile then I think it can be good.

S6: Claire smiths style, where she was doing about the different foldings and that,

What with the playdough?

No, not with the playdough, she was doing drawing.

I: Playdough?

S2: we had playdough.

S3: That didn’t go down too well,

No I wasn’t keen.

S3: Some people found it good, some people really liked it,

S5: I don’t know if its just me or, I just got really distracted and just started making little things out of playdough.

S4: I just felt like a child, it just frustrated me.

I: Can someone just give me a brief explanation of what the play dough session involved?

S5: It was for embryology, and the folding of the neural tube and the formation of the chorionic cavity and the amniotic cavity and how everything went in place and things, and she gave out play dough to everyone, and made us actually create the blastocyst or whatever, and made us change it into a the different layers,

S2: The next bit.

S5: and see them all move, I’ve forgotten what they were. But erm, but yeah, I didn’t find it, that useful because she had the images on the slides and we were just replicating what was on the slides. Yeah.

S6: The images were good but I don’t feel like actually doing it added anything more, like there wasn’t any new information to be gained from it, it was just , you were more worried about what colour playdough you were trying to do.

S4: I just felt, it was patronising, I just didn’t. I know everyone else was like, O, playdough its really fun I just didn’t think it was helpful, in any way,

S2: I just wanted to play with playdough.

I: I suppose there is a difference between it being enjoyable and you actually learning.

S4: I think also you’ve got to remember we have mature students in our lecture theatre and I think giving our thirty year olds play dough, I don’t know, it just doesn’t make sense to me. But that’s just what I thought. But I appreciated it, I appreciated, she was trying something different (agreement), and trying to think of a way to engage us so I’m not completely critising.

I: I know in the lecture I gave you I used poll everywhere, have you had that in any other lectures at all?

S*: I think we did

S1: Maybe once or twice,

S2: Or something similar,

S* I don’t think it was in this module.

S5: We do a similar thing in the imaging, when it works,

S2: It’s worked once,

S5: The thing is they hardly ever,

S3: Its either crashed,

S5: Or they don’t know how to use it,

S3: They don’t know how to use it, we don’t know how to use it.

S5: And instead of them working out how to use it, they open the website, they click on something, oh it doesn’t work, nevermind, let’s not use that.

S6: But they still use the images I think, with the actual imaging, because its all from the website instead of having it in a slideshow I then can’t annotate what is happening. I’m too busy print screening it to then put it somewhere so I can then put for future reference. I don’t pick a lot up in the actual imaging thing, because I am too busy trying to get something I can take away from it.

S3: it would be really nice to have a powerpoint of what we need to learn from imaging because I am not really sure what we’re meant to learn from it. And then explanations and things, have it in a powerpoint and then either, if we have to do self-directed learning, we can like annotate what the things are we are looking for. But at least, know what we are looking for. Erm and have things so you can annotate. Where as on that programme, taking notes is impossible, the only thing you can take notes on is whether it is D1 or D2, you can’t say what this picture or this picture. You can’t say where is was from or what bit of it.

S6: It’s kind of useful for revision but when you are actually learning it, its not that great because you can’t take anything away from it. Other than what you have remembered.

S2: I find that I can make notes from the imaging. (S1: I do to) I just write down which question number it was, and what was important about that slide ….. and the one that we had in the PC suite where it actually worked, I think if people had bothered to participate, it could have been good.

S3: It’s never worked though.

S2: …Of the like 60 or 70 people in the room only 25 people were answering each.

S3: That’s a problem with not the lecturer, that’s a problem with people wanting to go to the earlier session. So you have 60 people in one room, and there is only 10 in the next session.

S2: I was in the first session and there was still only, 25 people answering of a room of full of about 70 people.

S1: I think it did work though, the poll thing, go through the poll. Oh I got that one wrong, but now I know what the answer is, I actually did think it worked….

S3: I think it is a really good idea.

S1: I think we have had ….

S2: Claire smith used the poll last term and she had a few set up, this term but then didn’t actually use them I think,

I: when she has used them, how have you found them?

S*: I liked them

S*: It forces you to answer the question and actually think about it.

S4: I like them, again, if enough time is given, if too much time is given I just get a bit bored with it. If they ask a question and then leave the answers and you are sitting there for ages, I think if its like quite quick like, you’ve got 30 seconds, answer it.

S6: If it keep the lecture flowing so you are not disrupting the flow of the lecture.

S4: yeah and then you don’t get like chatter as well.

S2: I don’t like the ones where they don’t have a web option because you don’t know to text in whether it’s a number you are going to have to pay for. Or not.

S3: I just assumed you didn’t have to pay for it.

S2: you see I assumed, that you did because, anything you text in you normally have to pay for.

S3: I thought let’s just not worry about it, let’s just go for it. I was doing it on behalf of three people so 3 of us…

(discussion about cost)

S3: I think they’re good I think its good if. They are free then to say

S2: this is free.

S3: …its not going to cost you anything, you might as well participate.

I: Is there any difference using the poll app compared to the lecturers you have had say, ‘out your hand up if you think this, put your hand up if you think that.

S6: I think you get more participation with the app. It’s anonymous. But at the same time you are still finding out the right answer and thinking it through.

S2: If you are the one person who put the wrong answer, no one will ever know.

S5: It encourages people because it is anonymous but I think it also discourages people because it takes more effort, get your phone out,

S2: And also they can’t see if you have participated or not. Whereas if you have everyone put their hand up for different options then you can tell which one of us hasn’t put our hand up.

S6: especially if they don’t let go, like that Australian guy, if they see that not many people have their hand up, and say come on everyone has to answer then that will get everyone to do it.

I: I think today you were supposed to have a lecture with Dr Montgomery, where she asked you to prepare for it. I think she gave you all the slides and then, in the lecture she would not have used the slides, she would have done something else, something a bit more interactive, meaning you could have engaged a bit more and consolidate the learning you have already done in your own time and that is something, its got a label, its called the flipped classroom. Because you are doing what you would have done in the lecture theatre at home. Did you guys prepare for it? What are your thoughts on doing that.

S4: I think its quite a good idea because as you say, at home is when you do most of the recall, like trying to remember, anyway, and then, it means you can come into the lecture like, knowing what you want to get out of it in a way. Being like, I completely didn’t understand that some I’m going to to try and like learn and seewhat they say. Whereas when it is the other way around and you go home and you realise you really haven’t understood something, then you can’t…

S3: you’ve missed the opportunity.

S4: you’ve missed the opportunity to then ask them. So I think it is quite a good idea.

S6: I think it’s quite like MTs, for me, I get to do all the lectures see and then see what I actually remember and what I actually do know, and then I can actually ask questions as well. So I imagine this is a similar sort of thing, just on a larger scale. But erm, yeah I think that would be more useful.

S2: I disagree, again.. (laughter)

S6: I do too, its okay.

S2: I didn’t think that most people would read the lecture slides, although I did this morning, I did not think that most people would….. and skipping over a few slides bothers me, so not doing a whole lecture, its erm yeah, I don’t like it.

S4: I think it encourages independence though and independent learning. Because when we go into third year, we’re not going to be spoon fed, the lecturer standing, telling us everything we need to know, you have to sort of go and like learn things for yourself and I think its quite good to do. I wouldn’t want every lecture to be done that way but I think it is quite nice to do with certain, a few, just to mix it up a bit.

S5: But also, I like the idea of it, but I have a problem with it because no matter what you say, for me, it would be more work, because you are going over the lecture first, then you are going to this session to, explain it and then extra notes from that session which then you have to go over again. So you are doing three sets, three sessions of learning about the same information while,

S3: But wouldn’t that make it go in better.

S4: yeah I think it would be helpful.

S5: Its more time that I don’t have.

S1: I sort of agree with you S5.

S5: Although we’d like to do it that way I don’t have that extra time.

S1: I think it is somewhat a little bit optimistic because say we’ve got 5 lectures that week and then Friday we’ve got, that lecture. For me personally I’ll plan the time when I am going to go through different lectures, and erm its quite difficult if I don’t do one of those lectures because I then have to make time for it elsewhere. If I then have to make time before the lecture to go through it, its quite difficult to plan and get everything done in time so I sort of agree with that. But I think in terms of actually remembering the stuff it would probably work quite well.

S*: It would help.

S3: I normally wouldn’t have gone through it, I’ve never prepared for lectures before, I just walk in and sometimes listen. But I did yesterday because I happened to walk in on housemates, who were going through it so I was like, why not. So we sort of sat and we ended up discussing it. There was 3 of us and we went through the powerpoint. It didn’t take very long because we weren’t trying to learn it, we were trying to look at it before the lectures, pre-going through it. It was only like 37 slides long, which didn’t take us very long at all. And we ended up discussing it between us, and asking questions between us, and I got a lot more from that than I would have going through it on my own because we could ask like, what does bleeding mean. Is it referring to like, bleeding out og your down below or bleeding out of elsewhere, and discussing it that way. And then we, could sort of question and say what does this mean this is contraicting each other and blah blah blah. But from going through it before hand it hand meant that we had already thought about it and thought about questions that we want to ask, which meant when going into the lecture I would have been more engaged because I knew well I didn’t understand that so I want to listen to this. To see what the understanding of that is. So I found it quite, if we had the lecture, we obviously didn’t have the lecture, I would have questions in my head that I would ask about it and I would be engaged in that. Erm I think it is good to have that before and then have the lecture then and afterwards. I see it as opposite to you, I don’t see it as extra work, because you have to do that work anyway. You’d have to like be revising that lecture afterwards or, try to learn that lecture, so I think having that lecture before… I would spend less time doing it this way. But everyone is different like,

S4: Less time trying to learn it in the long run,

S6: It would probably be more useful to have that sort of thing at the start of the term. To get a foundation and to have actually properly have learnt that. Stuff you are going to have to build upon.

S1: That’s probably a good point actually. (agreement)

S6: The stuff that we are going to keep revisiting throughout the term, it is probably better to do it that way. Where you have gone over it 3 times, instead of waiting until the end of term where we already know that 5 weeks ago sort off thing.

S4: What I personally wouldn’t mind and I know some people might disagree, is not like to have set work to do, I wouldn’t mind them like maybe giving us a booklet or something to read over the holidays. Not that we have to do, not work we actually have to do, just, I mean we have a month off.

S2: like A-Level prework

S4: I wouldn’t mind having something so that I turn up, I know we can all do it if we want to but we don’t know what the module is expecting us, I wouldn’t mind having say over Christmas just a few pages to read, just for like the basics, so as then when you came, you felt like you had, like a foundation, a lot of people would disagree, but if it wasn’t a compulsory thing, it might just be nice to have something to read. (44.04)

S2: we don’t get the timetable until like two days before.

S5: we are given a reading list but then it is like 15 different books, and you don’t know which one to choose, is this too much. What do you need out of it.

I: Do you find that with lectures as well that you don’t know what you need to get from the lectures,

S3: Yeah, whats important

S4: sometimes with imaging, yeah.

S1: And the ethical ones.

S4: Imaging

S1: No idea, whats going on.

S3: If there is a little bit at the bottom saying, and what have you, one of the keywords because often with the markers is that they say you have to have the exact phrase, so knowing what will give you the mark in some ways, its quite an exams based sort of thought, but don’t know always from lectures what are the key bits. What is important. Cos there are some people that learn every single little tiny fact and letter to the detail. I don’t think I could physically or possibly do that. Erm like learning this and this and this. And then you go into the exam and you’re like I should have known that but then I shouldn’t have known that what is important and whats not important, so yes.

S6: And I think sometimes, they put outcomes but they’re just too vague, like we had, a 60 slide lecture and they have two points, and it was just so vauge and I’m like, oh I only need to know two things out of this entire lecture which isn’t true but it still doesn’t help point out which bits are important.

S2: Or sometimes they change the title of the lecture and ignore the learning points that are in the handbook completely and give you a lecture about something else.

S3: Some people like, at the end of the lecture, we were saying about the questions, some people do like questions some people don’t like questions. But maybe anyway just have questions, whether they are going to ask them or not, when you go through like your 60 powerpoint, having on the 61th slide, questions you should have got really, what you should have got from that powerpoint.

S5: Could have been 85^th^ Slide, this term, Anna Crown, 85 slides in one hour, she speaks very fast..

S6: It’s not as if they are sparse slides either,

S2: It’s the symposiums where they talk solidly for 3 hours,

S6: Oh yeah that was awful.

S1: I’m trying to think which symposiums have been really good. Cos theres been a that where…

S2: the loss of the baby

S1: … the loss of the baby one was probably... Or the one with the twins and erm, and he did the ultrasound of it.

S2: the twins wasn’t actually the symposium I think, I think that was the lecture afterwards,

S1: Either way

*Talking over each other*

S4: What I liked about that, he gave some cases again didn’t he, and was like would this be monochorionic blah blah blah, that was quite good because you actually had to think about it.

S6: I think that lecture overall was very good. He gave us the understanding with diagrams but then also enough information then to go back and read it, then he gave us case studies and allowed us to go through it, explain it, and then he’d come back to it again at the end with the ultrasound and that.

S1: and it was engaging, it got people, to like to hands up. I think he did the hands up thing as well, I think that works quite well for me anyway.

S3: I think a certain amount of, going back to the rapport, why we engage, it’s an element of the attitude of the lecturer.

S2: We like people who are nice to us. (laughter)

S3: If they’re nice, enthusiastic, cos some people do just like, they sound, bored talking about their own subject.

S2: We had one about brain surgery, which sounds really exciting and it was one of the dullest things we’d ever done this term.

S3: yeah, if they’re passionate about it, they’re confident or they speak clearly, its all sort of he normal things if you are giving a presentation they’ll tell you to do.

S4: Or if they are too far the other way like that recent, imaging one we had where he just came across so badly, he was very arrogant I thought, that was just my opinion, I just thought oh my gosh.

S2: I liked the arrogant guy.

S3: Like as I say I think some people did like him, and I think some of the principles he was trying to teach were potentially were very, very good for us. Because you can’t when you’re like telling a patient or explaining a case to a patient you can’t be all like oh I think it’s this, or is it this. So that’s fair enough to tell us that.

S4: That’s not the problem, I had with it I just thought, erm..

S3: buts it’s a lecture, its not now. You don’t have to be telling us we’re wrong to … it’s not the right environment to be teaching us those principles.

S6: I feel it gets my back up a bit, if they’re like you should do this, I’m like ‘alright mate’.

S4: and also, it’s the same thing when like quite a few people, he’d ask a question out to the group and someone would answer, and they;d be wrong. And rather than saying, no, but good thinking, he would just be like, er, no.

S*: Or why did you think that,

S4: Yeah or like, or like, why did you think that. There was none of that it was just like, err, no. anyone else. And I just found that a bit, disheartening.

S6: I think the principle he was trying to give made sense, it was just the way he did it.

S*: It just was not appropriate,

*talking over each other*

S4: What I thought he said was fine, he was just rude…. I wouldn’t want him as my doctor. But some people really liked it so…

S3: I think it is better than being floppy, like being all like ‘oo’ like much better, I prefer someone, that doesn’t probably make any sense,

S1: No, no I get what you mean.

S3: Can you put it in English for me?

S1: Where they are like, good try, but no.

S3: Or when they are kind of wavery, instead of being, this is this, this is this, sometimes it does work.

S4: I like assertiveness as well, but I don’t think you have to be rude, but then I guess we are going to come across rude consultants, so you can’t always have it.

S3: it’s good teaching for us in that respect but that’s not what we are trying to get out of a lecture.

I: How do you find, just a slides presentation, where it is just slides and someone is talking.

S4: it depends on how they talk really.

S6: and depends on whether they are just reading off the slide,

S2: and how, if they’ve got 80 slides to do in an hour and they’re have solid chunks of text or a few bullet points and a diagram.

S3: Diagrams are good,

S2: or if they’ve done them on black slides.

S1: The actual structure of the lecture, so some of them have like headings, where like okay, so here, this comes under this heading, this, this, this and this. And then, we’ll go onto the next bit… and then they’ll actually go through it, and the title will match.

S2: It makes a lot more sense later.

S1: a good person for that is Harry Witchel. Or Paul Gard maybe, in fact Anna Crown does that really well as well. But I find it much easier to go through it afterwards and actually during the lecture to follow it. So I didn’t get that but I can put it to the back of my mind and go onto the next bit. I can actually follow.

S*: Chunking.

S3: an overview as well, not an introduction, an overview, to say, this fits into here, this fits into here, we just get lost with what we were talking about. And I forget like, its clearly all this information but what is the information regarding to. Having the titles, subtitles heading, whatever you want to call them. Chunking it down.

S5: But not spending too long,

S*: No not spending 15 minutes on it.

S3: Objectives, I don’t know, there is a lot of introductory bits that I think are absolutely pointless,

Yes such a waste of time.

S2: I don’t want the learning objectives read out to me. I don’t care, I can read them.

S3: Yeah that’s fine, that’s just reading them, but doing the explanation and all the important bits, you actually.

S6: Maybe just having a sentence between each chunk cos I sometimes get confused as to which part of the body we are in.

S*: yeah

S6: if there are like, so this is the uterus, now we are moving onto the cervix. Oh okay then its easier because then I know where I actually am.

S3: rather than assuming we know…

S6: oh, is that only in that area, okay.

S2: the paediatric anatomy one we had, that was well split up. This is the head, this is the thorax. (agreement) And the one about, how to make drugs, I don’t know what is was called, with the,

S4: the hormonal one..

S2: yeah, how to make hormonal drugs or something, that was split up into, this is this bit, this is this bit.

S5: That was nice.

S4: I think ultimately it doesn’t matter what teaching style you do, whether you do flipped classroom, just a presentation, drawing. I think it is just, it literally is for me anyway the lecturer, like, if the lecturer is engaging, assertive, can keep, keep people engaged, so there is not like chatting going on, erm, and, speaks at the right rate, the right volume, then I think it doesn’t really matter, I think it depends so much on the lecturer.

S3: Volume I think is a massive deal, we’re young and we have average hearing, adequate hearing, not everyone in our lecture probably has as good as hearing as well. But sometimes I can’t hear them. They need to be much louder and clearer. Whether that the microphone…

S2: Sometimes you can’t hear them sat at the front though.

S3: which is ridiculous like.

I: Well what I wanted to find out is what teaching methods are best, what you are saying S4 is that is purely the lecturer. So its hard to change every single lecturer we have if you get what I mean.

S3: But I think also when they are anecdotal as well, and obviously a lot of these are doctors from the hospital, if they, even if it is not written up as a case study, I once had this one person who had this, it’s putting it on a person.

S*: It’s easy to remember..

S*: It puts it in context.

S6: Yeah, exactly, its not just a list,

S3: Pictures of what it looked like, pictures are good.

Labelled pictures,

S3: See, its like cushings though, rather than having a whole load of text. You have the text, but then you have the picture demonstrating it as well. It helps, pictures, diagrams.

S1: CC I like her lectures, (agreement) and the fact that she goes through every single person, asking every single person a question, cos we’re sat in the computer room, so she’ll go around each row. And I think that is quite good because, I can like, you can sort of work out when your question is going to come up.

S3: You’re engaged as well, you are forced to be engaged.

S1: It does actually engage you and I feel like I get quite a lot from her lectures,

S4: her pictures, her labelled pictures as well are good, (laughter)

S3: Demonstrating normal and not normal, because that is quite difficult with pathology to see what, what is wrong with this, ..

S2: When you don’t know what is supposed to look like to start with.

S5: I like her lectures but she does read off the slides, a lot.

S1: She does,

S6: she’s better in the computer room when got the she asks questions and then goes around.

S3: the advantage in the computer room is that is much smaller teaching groups as well, that’s a big advantage.

S2: I think one of the main things I like about her is she clearly wants to be there and she is enjoying it (Agreement)…

S4: she is really passionate about what she does.

S2: where as the lecturers who don’t want, who clearly don’t want to be there. Don’t make me want to pay attention.

S4: TW is really passionate as well, yeah, and very erm, if you ask a question he’ll actually, like, give you a long answer. He’s very approachable.

*Microscope sessions*

* turning up to sign registers and then leaving the lecture*

S1: I think a lot of that is to do with how important they perceive the lecture to be, because, of for example they said, alright for this lecture, everything on this lecture is going to be in the KT. Every single person would, everyone would pay attention to it. I mean, we’re all exam driven, so if they emphasised, okay like. I’m not saying like hint towards the answers but like, if they er, emphasise the importance of it maybe, because I think if people perceive it to be important they’ll

S2: I think some people just don’t care about lectures,

S6: and the thing is there is always Camtasia. I think a lot of people, rely on that.

S1: Yeah okay.

S6: And I mean, I can agree to some extent, why Camtasia is used, sometimes, like I said its useful to pause it, look something up. Because I am not getting anything more, its just I am doing it in one time block instead of two. So I can kind of understand it. I don’t miss the lectures because I am worried I’ll miss something, I can kind of understand that and then quickly sign in and thinking I didn’t waste an hour there.

S3: I know a lot of people have said like it’s pointless going to lectures because they don’t get anything from them, but that’s not everyone, that’s not a representation of everyone, that’s a representation of some people in the year. Like they won’t go or they’ll sit at the back and doing notes all the time. They personally think they won’t get anything from them.

I: And do you guys get things from lectures.

(agreement)

I: What is it dependant on?

S4: it depends very much on the time of the day as well I suppose, and when we have a 9-5 Tuesday, I know that 4 o’clock lecture, I’m really not going to get much from it. I’m just so tired and drained.

S*: sometimes your brain has given up.

S4: Yeah sometimes, but most of the time I think its worth, its just also like, I mean, personally for me, if I didn’t come to lectures, you wouldn’t be doing anything with your day, I mean it is like, you’re at medical school, it’s just nice to come in, in a way and actually do things. I don’t understand people who just want to stay in bed all day,

S2: Like, we are paying £9000 pounds a year.

S4: yeah, I don’t get it.

I: you were saying that you are exam driven, when you are in the lecture are you ever kind of career driven. Towards, I might need this when I am a doctor.

S1: Sometimes, (agreement)

S3: I think, I find that a lot in DR, they’ll tell us something, like pudendal nerve block thing. I’ll probably always remember what that is, because it is clinically relevant, and it makes sense of, okay I might actually need to know that one day. Whereas by the time we have got to the third reason for prolapse.

S2: you don’t care anymore.

S3: I don’t (laughter)

S5: In the end I mean, we’re all here to become doctors, not to become hardcore scientists,

S3: I don’t think some people are though, ~~~~

S2: And like a recognise that everything is something that might be important and something I might need to use, but I’m never going to get to use it If I don’t pass the exam,

S6: Yeah, unfortunately that’s the bottom line. Which is my problem, but they’ll be a few things where I’ll think, oh actually.

S2: This might be something really really important but, if I don’t pass the exam I’m never going to get to use it (agreement).

S1: Yeah but the best lectures I think are, for example the obstetrician one where he was talking about twin births in pregnancy. I was thinking, I could be an obstetrician, yeah this could be interesting. But then the next person comes along where I’m like, I am definitely not being that. And I think again, it is very much on how they present it. But even the lectures I get most out of, are the ones I can see myself doing in the future and I want to read about and. But for example the ethics lectures where that is just written off the slides and its just quite confusing, I’ve got no desire to read further on.

~~ethics lectures should be an Mt or something~

S3: I think an element of listening to lectures, A for me it is like, how easy it is to pay attention to the lecture, but also like in terms of, whether I am going to sit and chat or whether I am going to be quiet is how much respect I have for the lecturer. So for example if they’re not very nice or not very friendly, like the guy you were talking about before who was really patronising, I’ve lost my respect for him. You’re going to have more respect for, who is it, Dr, Dilly. (agreement) You just have so much respect and you’re not going to like talk. Or like be disruptive because like you’re who I want to be and I’m going to listen and like, I don’t know if that makes sense.

S4: No definitely.

S3: he’s enthusiastic and his voice was varied, it wasn’t monotone, he spoke clearly, his presentations had diagrams, it was clearly presented.

S1: well set out.

S4: his PowerPoints were probably the best to revise from.

S6: So well structured and so well set out. When I wrote out my notes I was just writing exactly the same stuff, there is no other way I can write this, its already perfectly set out.

S3: that’s the other thing like, what he is writing is clearly laid out. There are some lectures which are obviously, I don’t know if it offensive, it’s not offensive, they appear to be dyslexic, or that they can’t do English very well. What are you saying like?

S4: and like the sentences don’t make sense,

S3: … and its just so difficult,

S4: or they try to abbreviate things, but they don’t tell you what it actually stands for.

S6: I have to say abbreviations is one of the big problems that I have, when they say it so many times and I’m like, what are you talking about? And then I go back later and I figure it out or I ask someone else, and it makes a lot more sense,

S1: I was actually just thinking about that. When lecturers assume we know something and we don’t its very hard to understand obviously what they’re talking about.

S2: And no one will volunteer that we don’t know what they’re talking about, because no one wants to..

S3: I think that’s a big deal with us, we’re medical students, we’re much less up our own arse than some of the other medical schools, but we still won’t admit we don’t know something to a certain level, and I think that makes a big difference, so what the lecturer can do to help that is doing different things like put you hand up, rather than asking us specific questions, so but also making us feel comfortable and feel relaxed and chilled out, erm removing a bit of, inhibitions, erm, by being chilled or making a joke, bringing us on par with them, making us sort of like them, appreciate them, rather than feeling in a situation where I’m not going to answer anything because if I get it wrong I am going to look like an idiot.

S4: Yeah, its also nice when they say, is this pitched at the right level for you? Quite a few lecturers do that. They’ll do like a few slides and then say. Have I completely lost you all or is this easy, and you’ll either hear like a grumble or whatever from the audience, and most of the time that is okay, its just nice to have that opportunity to say like erm no I don’t understand that,

S2: Can you still hear me, are you following me do you need a break.

S*: any questions,

S5: I would have liked that question for the nuclear medicine one, (laughter)

S4: Oh yes!

S5: she rambled on about all sorts of different ions, and radioactive isotopes,

S2: Oh I had no idea what that lecture was about,

S4: It was so specialist, I swear it is what they like do for a specialist exam in like nuclear medicine.

S2: well some of the lecturers, they clearly use the slides for other things. Like they’ve got about 100, slides and they have just picked a few of their, standard set of slides that they show to loads of people, to show to us. We had a few people especially in symposiums who do that. But we had a few lecturers did that as well so.

S4: Yeah, I think the symposiums this term have not been as good as last term, I thought they were actually really good when they had like a lot of patients come in erm, yeah I think that is the best bit.

I: What is different this term?

S4: Erm I think A they have been a bit less organised, we’ve had a lot of people not turning up for random things,

S3: We’ve had a hell of a lot of lectures cancelled.

S4: we’ve only had two patients I think. And we liked having a patient in a symposium because

S2: it wasn’t just a lecture.

S3: It made it more interesting, and real.

S4: with the loss of the baby one, when she was talking about all the drusg she was on, the process and like, I don’t quite remember it off the top of my head now, but, I’ll always remember her, and it makes such a big difference, I think.

S5: we didn’t get the dog though,…

I: So how do you guys use lectures in your learning?

S3: go through them, reading from the slides, trying to understand the slides, some slides you have to read about 10 times to understand, and some you only have to read it once and you’re like I get that. So that makes a big difference,

I: Is that because of the content or just the way the slides…

S3: the way they are written. Because sometimes I go on Wikipedia and google the same thing and think, ahh that makes so much more sense, just because it is written in a better way.

S5: or terms are defined,

S4: Yeah, defining terms, quite a lot erm,

S3: So you’ve got the notes at the bottom as well and if you have stuff explained isn’t on the slide, I’ll then google that and I’ll tend to post it in the notes then I’ll go back through the slides.

S6: See I’ll just, re-write them into my own notes, I read all my notes and condense it all and think, okay, if I learn nothing else from the lecture, I should be okay, if I just learn..

S4: I find it so hard to do that, I always say I’ll make keynotes and then

S3: So I have stopped writing notes because, it’s a waste of time, I don’t look back over the notes,

S*: I do

S3: That’s if you make good notes, then you’ll over them. But it depends on the powerpoint and my handwriting is awful I can’t read it, it makes no difference. Then I keep reading the slides, and I’ve started doing quizlet.

S1: Oh yeah.

S3: it just takes time. So I think, what I was thinking for this term is to read the slides and then from the slides just make questions, on physical paper itself.

S4: yeah that’s what I do.

S3: and then, when I want to go back through that lecture, I’d read the questions, if I can answer the questions, saying like what is this thing, and then I have to have three points about it. So then if I can’t think of three points then I’ll either google it or go back over the lectures,

S4: Yeah I do that with all my revision, I have this app and I make like questions, cos like, it just, you can then, confirm yourself if its gone in. what you’ve written.

S5: Sometimes they take so long to make but, you don’t have time to actually test yourself, but I think the fact is making them.

S4: Yeah, actually making them.

Yeah making them physically does,

S2: I write then I read, then I write, then I read, then I read some more.

S1: I just write them out. Yeah I dunno, I guess it obviously differs for every person,

S3: Yeah some people can literally just be, one of our flatmates he can literally just read the powerpoint and he’ll know it.

S4: It will be in his head like… but yeah just everyone is different aren’t they.

S2: I find it better to remember if it is in my own handwriting, because I can picture my own handwriting. I can remember writing it out so sometimes the order I wrote it out in helps.

I: So would you say you don’t learn much in lecture, its more ..

~~talking over each other~~

S6: you see for me, I learn most of it at home, or in the library in revision week, and I know it feels like I’m leaving it late, I must have something going, because it is not completely alien to me but, if you were to quiz me on most of it now, I wouldn’t be able to tell you very much sort of thing.

S2: I’ll remember it from the lecture until like the end of the week, but then if I haven’t done it again by then, I wouldn’t be able to tell it back to you but I’d recognise it if I saw it again.

S6: Yeah I’d recognise it I just wouldn’t be able to come up with it myself.

I: Learning in that way, does that have any impact on you in say, a couple of months.So say some of the stuff you learnt in neuro,

S6: it depends what it is though. If it’s clinically relevant I’m more likely to remember it.

S2: I remember more of the useful things. Than I remember of the things I thought, this is really boring, and I can’t see how I am going to use this, like I can’t remember the krebs cycle.

S3: I tend to remember things that link, things that make sense and that are concepts rather than plain facts I think next term is going to be horrific just learning all the muscles and all their positions> if I’ve got a link, if there is understanding, if there is knowledge behind it than that is much easier to remember.

S4: that’s why I quite like doing, I mean at the moment doing, with the anatomy society we’ve ben doing peer teaching, like mock Vivas for first years and that’s like so useful, at first I was thinking oh god I’m going to have to put in so much effort, but it didn’t actually, it surprised me how quickly I did remember things, even though I didn’t remember it to recall, once I’d read over the DR notes from last year again, like I did, it was there, in my head somewhere.

S*: You just need to refresh it,

S1: That’s what I feel with the lectures as well, you go, if I actually attend a lecture and then write the notes on it, when I come, first of all it will be easier to write the notes because I have already, got the understanding, I won’t really remember what is in the lecture, that they’ve told us. But then if you go back, and I’ve already got the, I’ve already worked it out in my head. I won’t necessarily remember it. I will have just worked it out in my head and that’s the same, for when I go back and revise it, when I read it, when I read over my notes and I write some more notes, I’ll be able to think okay, actually I’ve worked that out before, so it’s much easier to work it out and remember it, that way. So I guess, by going to lectures, it helps, in fact if I wasn’t going to lectures and just write notes it would be, I would remember less, or understand less. Than if I was to just write my notes. (52.07)

# Focus group 1: Flipped Classroom Reflections

S1: I thought the flipped classroom approach was actually very useful for my learning. Having gone over it before I was able to get a general understanding of the lecture and identify areas that I needed to explore further. It was also a good recap.

The content of the flipped classroom was slightly different to that in the lecture. This helped me draw links between what I did know and what was being taught which no doubt helped me remember it. However, I was slightly unsure as to what I needed to know. I also found that it was not too much extra work to handle.

S2: I did not find the flipped classroom particularly useful. I felt that all of the information covered in the session was included in the lecture notes, however there seemed to be more of tendency to get off topic than there normally is in a lecture and so we did not cover all the information included in the lecture notes as we ran out of time. I also thought that I didn't not gain any extra understanding of the subject even though it required extra time on my part because I had to go through the notes beforehand. I prefer regular lectures and I do not think more sessions like that would be a good thing.

S3: I overall liked the flipped class room style of teaching, but would probably choose to do more traditional style teaching, using powerpoints with information on regarding on what is said in the lecture. I feel this works because then when we go over the lecture, we can relate it back to what was gone through in the lecture and the information we take down and write as notes during the lecture from what the lecture is verbally saying, match with the powerpoint.

I did however like the fact that I went into the lecture having looked at the powerpoint before. This wouldn't have happened unless I had walked in on flatmates going through it and was encouraged to join in. However there was too much time between going through it before and when the lecture happened, so I had forgotten any thoughts I had had or questions I wanted to ask. In an ideal world I would go through lectures before hand, so I have a rough idea of what they are talking about and then during the lecture I am engaged and want to ask questions. I don't feel this would happen as there are so many students in the lecture hall and only the front row in reality is truly engaged. Lectures that make it work, are ones that walk around the lecture hall and force the people at the back to be engaged.

S4: I actually found the flipped classroom a refreshing change to normal lectures. It was engaging and we were less likely to 'switch off', because we had to create the information, and it was interesting to see what our peers had to say.

The only changes I would make would be:

1. Have the information typed up on the big screen, rather than handwritten on the flip charts. This is because it was very, very difficult for the audience to read

2. Have less information to get through. There was a lot said in the hour, and in fact Mrs Mongonery ran on longer than the hour. This meant that the 'scribes' struggled to keep up, and in the end they were looking a bit bewildered at what points they should be writing down.

S5: I recognize the value of the flipped classroom approach in that we didn't have to use any valuable time going over any information that was straight forward and easy to understand and people had more opportunity to ask questions and get clarifications for any part of the lecture they were unsure about. Also, the flipped classroom approach allowed us to expand on the information that was in the lecture and the more thorough explanations and further background information that this approach provided helped us build a better foundation for understanding this information. However, I also felt that this approach did still have a few downfalls. The one aspect of this approach that bothered me the most was the session's lack of structure. The fact that the session had no clear structure and was just aimed at answering people's questions meant that there was no motivation to move on from one topic to the next and consequently I feel we spent too long on certain questions and didn't get a chance to receive any clarification on the later parts/last few slides of the lecture. Furthermore, instead of using the lecture slides Dr. Montgomery had two students write notes on big pieces of paper at the front of the lecture theater which not everyone could see because of small writing and the angle at which they were placed in proportion to where some people were sitting, a problem I feel, could easily have been corrected by typing on a projected word document or investing in a SMART Board for the lecture theater. It would have been better to have these notes typed out, moreover, so that a digital copy could have been shared with all students.

So, contrary to expectation, I liked the flipped classroom approach, recognize its value, and wouldn't mind it being implemented more often if these two concerns were addressed. I do also recognize, however, that a flipped classroom session is practically useless for any student that hasn't previously gone over the lecture slides and therefore I worry that if used more often, the day a student doesn't have time to go over the lecture slides before a session - they will waste their day sitting in a room listening to information they don't have the foundation to understand.

S6: I found it very useful throughout – I went through it with a friend previously and we managed to work out the majority of the slides. I found it really useful to go through the knowledge to see what I could remember and also little anecdotes which made things easier. I think it would have gone smoother if the session had more structure to it as I felt we lingered on aspects too long which had previously been covered. Although overall now revising I have found that I remember more of the information and could go in with questions so come revision time I didn’t realise there was missing information or things I still wasn’t fully happy with. I overall I think it went well but was a bit difficult in the large group but still a useful way of learning – I felt that I actually knew things whereas in other lectures I find it difficult to actually learn the information.

# Focus group 2

I: So first of all I just want you guys to tell me about lectures, this term in module 203 that you’ve really learnt a lot from or enjoyed and kind of engaged with.

S2: I don’t mind starting, because one just popped into my head. I don’t know if people will agree, we had two lectures from a consultant I think he was the Australian one, and I am not being biased here, but he was very engaging and very interactive, and actually felt like I, it felt like he was pushing you cos he said oh come on you know, you have to answer some things. But you do, I feel like I learn a lot that way.

S4: The thing he did was use case studies rather than just saying you have to learn this stuff. He went through cases using examples and I think it really helped me think about what he was talking about rather than just learning about stuff.

S6: He made it quite interactive didn’t he (agreement)

S3: and like he wasn’t prepared for us not giving and answer, he was committed to having the engagement, to drawing the answers out. Yeah, it made you kind of switch on which is good.

S5: Even if you got the answer right he would still challenge you on why you thought the answer was right so it wasn’t just you would give him an answer and then he would get off your back, he would want to know your reasoning behind it. Which I thought was pretty good.

S2: yeah, that’s a good point.

S5: I enjoyed that.

S1: Yeah no he is a very good lecturer, he was the same last year wasn’t he, he was really good.

S3: he was stand out last year.

I: So what did he teach you this time around?

S*: ovarian cysts. Ovarian cancers.

I: Do you think your enjoyment was influenced at all by the subject matter? Or was it more him as a lecturer?

S2: No,

S1: Cos we all do all sorts of cancer of this, cancer of that, and different disorders. But there is no disorders of other things that have stuck into mind as much as his disorders of the ovaries. Does that make sense?

I: Yeah

S6: sorry I was going to say, because I don’t usually like that approach, like we have with the imaging lecturers I don’t like that at all. They really kind of put you on the spot but I think he was so high energy that’s what I really liked about it he was really high energy, he was really enthusiastic about what he was doing. We’ve had a couple of kind of going off slightly but I’m sure we’ll come back to him. A couple of other lecturers who have been like that. The erectile dysfunction lecturer was just like a stand-up comedian she was really funny. Then we had JQ who was kind of similar he wasa bit like a stand-up comedian too. And there was just so much, so much energy there. That’s what does it for me rather than the kind of questioning approach but the Australian guy was great.

S1: As long as they keep it interesting because if you are like, if they’ve kind of got the energy keeping it like, doing something funny here and there. Then it’s like ah that’s funny, I’ll listen to this guy. Where as if they are just like *drone* you’re like, ah I might just see what ASOS has got on at the moment like. So it is just like keeping it more interesting.

S3: What is similar between all those three is that personally I felt like, they were talking at me personally even if perhaps they were being very like general. And their language was very much directed at the audience rather than being all about what was on the slides, it was much more in the moment rather than preplanned.

S2: You know they were looking for an interaction with you rather than just delivering to a sort of nothing faceless audience weren’t they.

I: and did you come out of those lectures remembering what was taught? Because is there a difference between you actually enjoying it than

S2: I think I would remember more, I mean I don’t really remember much from lectures to be honest I find it the worst was for me to learn anything.

S4: his approach was like making you work through problems, it was like it made you think. I remember thinking rather than just passively taking it in. That helps me.

S2: It does feel like it goes in a bit deeper when you apply it

S4: You remember thinking about it

S2: Yes absolutley

S1: Also just in the sense of being engaged, if a lecturer doesn’t engage me then I will completely switch off…

S2: Yeah that is true

S1: … like I am sure X has noticed that I just, I will just sit there and do absolutely nothing so I will come out of the lecture and I won’t even be able to tell you what it was about whereas I could tell you what this guy was doing and that sort of thing so it actually does make quite a big difference. Like even if you wouldn’t necessarily know, maybe someone else wouldn’t come out of it and remember as many details, from it, but you actually, I remember more in general terms because you are a bit more interested.

I: So did he teach you with just slides and then standing at the front and then talking at you?

S*: yeah but then he had like questions as well.

S4: The slides were just very minimal slide of a few lines of text really and he walked around and he..

S2: Yeah

S5: I think it has been kind of a general rule, whoever uses that long stick, tends to be quite an animated lecturer,

S6: yeah that’s true.

S2; I think the worst lectures I remember some from previous modules is where you have got loads and loads of text, just a ridiculous amount and then maybe just pictures you can’t really even see the details of and you just feel like you are just being, they are just running through a big script, and it just gets, you feel a bit

S1: It feel a bit rehersed

S2: Yeah you sort of feel like they are not interested, so you’re not interested, so you don’t really learn anything at all.

S6: Or they might be interested in their subject but it seems like they are not so interested in teaching you. Actually that is true yeah, so sometimes you feel like, oh they really are interested in teaching, and they have thought about how this comes across, and how they need to change it so it comes across well. Sort of thing…

S3: I think when there is a lot of text on the slide as well it means that most of the audience are so focused on getting down every single detail on that slide that they are not even listening to what the person is saying. I mean when I find I have to make notes they have to be complete or I am not happy. So I will literally be scrawling as fast as I can, not listening. And that, and you can’t really get any engagement with that and you’re disinterested because you are just focused on the writing not on the listening so.

S4: so yeah, I think the amount of text on the slides is quite difficult for lecturers to get. So you want to get enough text so that people will follow the lecture, either before or afterwards, during the actual lecture there should be less than the maximum there should be. So you can listen to what they are saying rather than focusing on the text. It should be enough so that you can follow it independently but also less, than too much in the lecture that you will get distracted. There is a balance.

S1: They always say in SSCs and stuff you can only have this many points per slide and this. And then you go into the lecture like, oh it was nice to see that you stuck to your guidelines.

S*: that’s so true.

S4: But then that is kind of useful afterwards where reading it back..

S1: But RG he does his slides and then he does notes to go with the slides, so people, it’s quite easy even on a powerpoint they can add in the notes underneath, in the little notes bit. So then the actual slides are fine for you to keep after so you can see and listen to what they are saying and also, see what kind of the outline is of what they are saying and then after the lecture you can look back at the notes and say so that was that in more depth. And that was a bit more about that.

S*: definitely.

S4: Just thinking about it TW his lectures, all of his lectures are slides, of cells. In the lecture it was good but afterwards you sit at home like, what’s going on, it’s just another picture of cells.

S6: so that’s the tricky thing, he uses the laser and TW uses the laser quite a lot, you don’t see that on Camtasia on the slides afterwards and you can’t remember what, and if you look at this area over here and then you are left looking at the slide and thinking oo I missed that.

S4: Its just a balance isn’t it.

S2: He’s quick too, isn’t he TW.

I: so what lectures, have you had any lectures that haven’t been just slides and powerpoint. Slides and someone talking at you.

S2: Erm no. We were meant to have the flipped one with JM.

S3: A couple of occasions they have got the flipboard out. They’ll do a diagram…

S*: Claire smith…

S2: Yes CS does a different sort of stuff.

S*: Camera

S*: Playdough

S*: AC

S1: yeah, but I remember last year when RS spent a whole lecture on the flipchart. That really pissed me off. Because I was like, I’ve got the notes here, why are you making me trying to do this, especially when the flipchart. He might not have done it this year. With the flipchart, because the way the lecture is put out, the lecture room, if you’re sat on the side, you can’t see and I remember like one time they were doing the flipboard and I was like oh yeah I can see it and then , oh you can’t see it over there, now I can’t see it. So the flip, it would be a really helpful if they just got a whiteboard on the wall, like next to the smart projector, because the flipchart is just like…

S*: What does CS do?

S* she has a magnifier doesn’t she.

S6: yeah and that sort of works

S1; an overhead projector.

S$: it’s a camera going to the actual projector as well.

S6: it looks good,

S2: I don’t actually learn from those plastacine things myself, it doesn’t do anything for me.

S1: I think if I was like doing it at home and seeing, I find the easiest way for me to learn those things is YouTube animation. Just like watching it, like oh okay, that’s how that goes like that and

S2: same

S1: So I guess playdough in that sense is doing it yourself. But then you have got to roll this bit out. Instead of actually like moving it into the next thing you’re like oh no I’m going to take this bit out and shape this instead and put it back and then oh no, that didn’t really work out.

S2: it was quite disjointed and doesn’t look like it should.

S1: it’s a good idea to get it all kind of involved and that kind of thing but I don’t know how well it works necessarily.

I: so what was the camera you were talking about that CS uses?

S*: some new-age overhead projector.

S4: she has it there plugged into, her desk and there is a camera and she can like use project, like on the projector, like a live camera feed of what she has in her hands so either playdough, or skulls,

S*: or even just drawing out a diagram

S4: she sometimes uses it to draw out

S2: She is good at doing different things

S4: Sometimes yeah, sometimes useful, sometimes not it depends how she uses it.

S3: It depends what she is drawing

Sometimes the

S3: That is only as good as the person drawing it on the machine

S1: But then again the fact that doing that, I find it is quite useful seeing it drawn out from one stage to the other cos then you kind of see instead of just having two images that are already like on the board you kind of see how it goes from one to the other but then again it is some of the same concepts as the flipchart. The way that she does it is so that everyone can see it. So it is like having a whiteboard on the wall. Instead of

S3: I think it is quite nice in principally because it breaks up the pace of the lecture. It’s not all just slide after slide after slide it’s a little bit of a break while you set it up so that people can sort of take time out from scrawling and refocus.

S1: Catch up

S3: Yeah, its just a nice change of pace, change it up

S6: it does feel like she really does think about how we are going to take something on board and she tries lots of different things, I mean she brought her kids in last week for paediatric development and I really paid attention throughout the whole of the lecture. I find it hard concentrating for 50 minutes at a time but I felt like my attention was fully on what was happening and I think she worked quite hard to plan that session and bring it to life.

S1: She is very good at keeping things as kind of interesting as possible and trying to keep you interactive like in a different sense to how the ovary guy did it but still in a way that kind of keeps you looking either more kind of hands on or more looking. I mean like bringing her kids in, that was like a really good idea I thought. I was like ah yeah okay I can see her head is bigger than her head, but no it was good.

S5: It is very obvious she puts in a lot of effort whereas the ovarian dude as he is now known it probably comes more naturally for him, like his personality, it’s probably erm, but with CS it is obvious she puts a lot of thought into her lectures.

I: does that keep you more engaged, just knowing that she is trying?

S6: yeah I think so.

S1: I guess so. It makes you, if you know they haven’t put in any effort its as if why do you expect me to put in any effort but if you can see they have put in effort. Ah okay I am going to watch and actually pay attention cos you have put in the effort so it is the least you can do.

S5: one of things that is a big giveaway for people is, that haven’t, that could give a bit more effort is non-updated slides, that have been up for like 2 or 3 years and as they are going through them they say oh I’ve changed this last night but I’ve got to update your version, and 60/70% of the time we don’t get the updated version so you don’t make then notes then, then you miss out on the changes.

S1: and also if they are doing something, and they say I didn’t realise that slide was next then its like oh you did prepare for this okay. You didn’t even look over your slides, that’s nice. I know that a lot of the people are clinicians and they are busy and stuff but they have essentially said that they will dedicate some of their time to teaching so you kind of feel like you would put a bit of effort in to even look over your slides the night before and say oh, okay, that’s this that’s this.

S2: I mean that sort of connects in with the, I find it quite weird how we have to use lecture notes for our exams, rather than seminar notes or, I don’t know, I think sometimes it’s a bit odd you look at some of the lectures and you think, what exactly am I meant to learn from this, you know, because it might be 50 slides long and you are not really sure what is important or it does feel a bit like they’re not accurate. And you are not really sure whether they are the right thing to study from.

I: So how do you, how do you all learn from your lectures?

S1: I’ll go on the student central and they didn’t, I don’t know if they have done t as much recently but I know that in the first year ones they had learning objectives for each lecture. And that was really useful was seeing the learning objectives. But then for a few of them it is as if it’s said the learning objectives but instead of actually being the learning objectives its actually this lecture will cover blahblahblah. You’re kind of like What’s the actual learning objectives, it’s useful when it says like, to be able to explain this or to be able to describe this, that kind of thing instead of just being really like ovaries. Uterus. When the learning objectives where there, they were really useful. To look at them, before going over a lecture. You can kind of see what, if, you are leaving things until the last minute which I would never do, it’s nice to know what you will definitely need from a lecture. And what you can kind fo be like, ah yeah I will just leave that. Just as long as I know this bit first.

S4: it’s also really important about how they are phrased and how they are worded. Not just like a last minute thing they have added on to the end of the slides. It really helps you understand what the lecture is about. They need to be more succinct and to the point I think sometimes.

S5: HW is really good at that.

S6: He is brilliant.

S5: And also to the point where he puts, so if you miss, or if you look at Camtasia for one of his lectures, you will notice that alright he has got the slides up but he is also reading from the notes that he has got at the bottom, not the slide. And then he does that throughout. Also with, having the learning outcomes and the summary at the end

S6: and the questions..

S5: Yeah I find,

S2: Didn’t he used to have things with extra asterixs for the really crucial things and green boxes for things that were just extra.

S3: His structure is really good, some lecturers have a habit of jumping around a little bit, whereas he is very much this is this, this is this. You can, it’s much easier to understand something when it is all parcelled up correctly,

S1: it’s quite interesting as well though because he seems to have quite a slow manner of speaking, but he gets a lot covered. Whereas a lot of people where you think oh my gosh they’re speaking so fast, how am I meant to keep up, but then you kind of look at the end and think oh there wasn’t actually that much there but they seem to have set it all at a hundred miles an hour. Whereas HW he goes at a pace where you can keep up and write the notes and stuff and at the end you are like, wow I did all that.

S3: Yeah, some people talk a lot without saying anything.

S2: he sort of summarises a whole concept in a sentence where you can just understand straight away.

I: So JM was going to do FC with you yesterday. Do you all understand what is meant by the flipped classroom as a concept?

S2: I thought seminar.

S4: A seminar type thing where you do reading and then discuss a concept in a lecture.

I: So in a lecture they’ll give you, I think she gave you the slides, and you go through all slides and you have an idea of what is going on, so that when you get into the lecture you can ask some questions on bits you don’t understand. She’ll do some tasks with you. So I suppose more to consolidate your knowledge rather than having first contact with it.

S4: I think it is a really good idea and they should do it more. As long as you get the right material beforehand. It’s not too long, I think whats it, to orientate yourself would be really useful. I’m thinking of the embryology lectures with CS, I couldn’t follow them at all, I didn’t do any prereading and they were just an hour of me doing nothing, I would have benefited from prereading I think.

S1: I guess that is kind of our own responsibility. I always say to myself oh I will look over the slides beforehand but then I am too lazy to actually ever do it. And then you go, oh yeah I should have done it. It’s like the same thing in the DR, if you look over the notes beforehand it is a lot more useful than doing it yourself. It is a bit like, oh, okay we are kind of old enough to say, yeah but we do need to look over this beforehand.

S4: It’s true but I think they could focus the prereading material more, in a better way. Rather than just give you slides, cos if you look at the slides you are like, ah, what am I meant to make of that. I have to spend hours going through a chapter in a textbook, you could give like a 10 minute brief recording, I don’t know.

S2: I don’t know why they, you see I’ve studied before. Well you got given a pack of everything you had to know I mean it was law so you had cases, or chapters that were photocopied and you had lectures which were very broad and you had seminars to really get to grips with the lectures, is there a reason they don’t, is it because of the heaviness of the timetable in medicine they don’t do that? Is it the time it would take.

I: It’s more the volume.

S2: because it seems like the flipped, the lecture becomes the seminar but with a massive amount of people, is that the idea sort of thing?

I: yeah it would be, so the flipped classroom originated in secondary schools, so it was originally a flipped classroom. But it is talked about a lot in higher education, people seeing if it would work in a lecture theatre. That’s why I am interested in what your opinions are on it. The theory is that you could get the best teacher in the world giving you a 10 minute YouTube video and how HW does podcasts and stuff. You can sit there for 10 minutes before going to the lecture, pause if you need to…

S4: that would be really good.

I: but then I suppose, the other thing that you need to consider is if you have seven lectures on a Friday so…

S3: the volume makes it slightly unrealistic. Also given that our assessments come fairly thick and fast as well, especially with JM being on Monday, the fact that we had an assessment due Tuesday, and the Viva today,

S*: and SSCs..

S3: 3 major things we are working towards already, the lectures aside, if you are then expected to do additional work before the lectures happen it doesn’t given you time to focus on the things that matter. Necessarily, and so, the lectures get lost.

S5: I think it would be nice just at least, well I guess it is in the handbook, to have the outcomes of, this is what you will have learnt by the end of today, as a starting point, Maybe if you just start thinking about, then you know where you are slotting the information as you are getting it in the lectures and you are in the right place.

S1: Cos there are a few lecturers that do that AD is one of the people that does that. (22.40) he’s my fav lecturer of all time.

S6: he’s no that interactive, It’s his energy, he’s thought so carefully about the structure of the material.

S4: the way he phrases it as well.

S6: His timing is absolutely fantastic.

S1: I just remember the first time in a lecturer I saw him and I was like oh my gosh, this guy has so much energy, I do not have enough energy right now to watch this guy. But after a while, I will now like, if I am thinking I don’t know if I can be bothered to go this lecture and then I see it’s one where it is AD I think ah, okay I will go then.

S3: he’s also clearly preplanned it because his slides, just looking at the after never seeing him present are fairly hard to follow but yet he knows exactly how everything works together, exactly what he is going to say next. So he has clearly done a lot of work, behind the scenes to make him as fluent and fluid as he is. And good I suppose. So, you know, it goes back to the planning aspect, it’s really important.

I: It sounds like some lecturers we can all agree, they are just good lecturers they’re naturally good, but then I suppose the other thing is kind of what will make a lecture better when it’s just a bog standard, average person who has been asked to come in. And they are there with their best intentions, not the most charismatic in the world but, what will get you guys engaged and listen and make it a better learning experience?

S1: Just being enthusiastic I guess, like if you are standing behind the podium thing *drone*then you are kind of like, oh, just do something. And also just like, I don’t know, getting a bit of energy in them.

S4: for me it would be the pre-reading or pre-video or whatever. I could watch and then think about what I want to get out of the lecture. Have a look before hand.

S3: For me it would be if the lecturers persevered with asking questions, occasionaly they will start, they will get a wall of silence, they’ll shrink behind the lecture and not really ask anymore questions, but if they let that awkward silence hand a little bit someone will jump in because they won’t be able to handle the silence and then it is all broken and then it is fine. And then the interaction starts after that awkward silence. Even with the ovary guy it was kind of awkward at the start but then he insisted that we interact and we start

S2: yeah it is true, it triggers (25.24)

S3: it’s almost perseverance I would say.

S1: and also asking questions, you did the like poll everywhere and that was good but then also the time that, that takes can also be saved by just asking us to raise our hands. Ovary guy was like, okay what are we going to do now, we can do conservative, we can cut them open, we can do laproscopic, What are we going to do and like then ask people to raise their hands for different bits, and that also kind of gets you thinking, what am I going to do and then like they said instead of them just saying okay so you raised your hands for that actually asked someone why have you raised your hand for that, what is the reason behind it.

S2: It completely engages a different part of your brain or something rather than just listening. And I did the like the case study, I think whenever there is a case study or they talk about their own clinical experience then I always think oh wow a patient. Some they actually do, this is what it is for. I always sort of remember that story or whatever about that patient so that really helps me. Definitely.

S6: For me I think it is partly the lecturer, but the way our course is it is so assessment driven, it’s also the quality of the slides. So I think AD is brilliant, I love his lectures, but if I miss one of his lectures I wouldn’t be confident that I could catch up from the slides. Whereas although HW is great I feel like if I missed his lecture, his slides are also great. So for me, during this course, I’ve got a different experience as well like

S2. With this course the slides have to be 50% of the whole experience. They have to be really good for the KT and then it is just nice if the lecturers made it really interesting and clearer and thoughtful as well. That’s important too, but a definite split for me.

I: How did you find using poll everywhere?

S1: I thought it was good in that in the beginning though I was like, okay then if I send a text. I was like, oh actually that takes a bit longer and then kind of for the next time I had downloaded the app and then that was like dead easy. So I found the app was really useful, the texting bit wasn’t as useful I was like ah yeah what’s the number.

S2: I did that as well and I sent it to the wrong number.

S1: yeah, so, I think, I guess using the online bit would be the same as using the app kind of thing. Doing it that way. But I found the app to be useful. Also I think, the one that, I know it’s kind of difficult, it’s a different system but when they do, when once upon a time the imaging lectures worked and you could answer things then okay and see what you have put, and then you could see peoples answers without having seen people put it before.

S4: I had no idea that worked before.

S1: Once upon a time it did.

S1: whereas on the poll everywhere you could see what people were saying and that might influence your answer.

S4: I find generally the radiology teaching, the lectures are good for me, the teaching is good, the resources are really terrible. You can’t use the website, there is no actual diagrams or what you need to look at. So going back over it is really frustrating for me.

S1: the one time it worked it was good.

S4: it is 10 years out of date the website they have got. It’s buggy and

S2: and as you said, when it comes to exam time I always think, what do I need to learn? How do I actually review imaging? It’s just really bizzare.

S4: It doesn’t work, it is not functional.

I: I think it worked when I was in year 2, black and flurocent green interface. Why isn’t it working this year? Is it people don’t know how to use it or.. it’s just…

No it’s just the system

*explanation of how the system should function*

They could see as a group how well we did.

In the lecture theatre when it is dark and they are just handing around a lazer pointer.

S6: what do people think about going down to the front, because the guy who did our imaging lecture this time didn’t say use the pointer so much, he actually got people down to the front. This was last week, did you go?

S2: at least they did go along the first two rows, I’ve done about 3 thatt way.

S3: wasn’t that a bit time consuming though? If they’re

S2: It was a bit, it was a bit.

S6: I’m not sure how comfortable I would have been.

It was a bit shaming

S4: It was volunteers,

S6: No it wasn’t

S2: He picked on them, it was a bit shaming.

S4: he asked them if they would come down or not and

S2: as if you are going to say no.

S4: I don’t know it would be useful to have like a deck of images, with the answers on that you could look through.

That would be so useful!

S2: really good idea.

S4: arrows and, it would take like 10 minutes. Maybe a bit more than that but then it would be done.

S2: because we don’t really have a recommended text book, there is not really anything like that so if you could add on a handbook that had all the modalities in the front and how to distinguish them

S5: there is a wealth of resource there with the imaging but it’s almost as if it is locked up in a safe and you can’t get to it with thath website so. So trying to make it something that doesn’t rely on it, i.e. works I think that would be an awesome idea.

S3: we had a thing called question of anatomy, in my first year.

S1: That is so good.

The online quiz,

S2: I loved that, I used to do that non-stop.

S3: And they had quite a lot of the imaging as part of that. And that was really useful.

S2: and they got rid of it.

S1: no it’s cos they didn’t do it on purpose though, it’s the people that ran whatever it was they closed it down so it wasn’t them who got rid of it.

S2: I loved it, it was brilliant for revision. It was and it was just a little break almost where you were still learning. When you were studying. It was like, oh, I’l do that for a bit.

S1: It was like where I would I do it for hours on end and be like I’m revising it’s fine! Not actually doing any real revision. (33.12)

S3: it was really useful.

S1: yeah that’s a shame that has gone.

S5: Back to the lectures and lecturers, or lectures vs lecturers not to be mean but the lecturers only really got themselves to work with so you’re going to get dry ones, you are going to get really funny ones, energetic and not so but I think the one thing they can all control in the lectures is the slides and that is why I think it is really important to get little things like learning outcomes, too much on there and possibly also putting what they are talking about in their notes, would be a really good way forward. Yeah I can’t think of any other way to..

S6: They are given all those guidelines aren’t they, some of them follow them some of them don’t. They are told how many words to have on the slides they are told how many slides to put up to and everything I think, but it’s variable isn’t it.

S1: Also it can be very daunting going into a lecture thinking oh god there is 80 slides in this lecture and then it is like, they are going to have to spend less than a minute on each slide, and then you are thinking oh gosh, how am I going to do this.

S5: 30 seconds a slide.

S1: I mean some of them might be able to do it but just generally.

S3: It’s not whether the lecturer can do it, it is whether I can do it with the lecturer, that’s more important because it is not them delivering it that is important it is us understanding what they are delivering at the end of the day.

S1: that’s where 2 lecturers had their differences last year.

S3: it’s interesting because I found the very fast, machine gun approach almost better than the repetition thing because I just switched off. If you repeat stuff too much.

S2: the concentration just goes, doesn’t it.

S3: If I get it the first time and you reiterate it like three more times I’m gone. I’m on a different plant.

S2: It’s true, you feel insulted almost. I get it okay.

S3: I think some of like the best lectures have 30 minutes, 40 minutes content and if you need more time to explain because you don’t think that people are getting it you can use the extra 15 but if it ends early then that is fine as well. It’s not a problem to finish early if you are prepared to use that time if it needs to be used.

S1: it is difficult when we have a full on day of lectures 9 til 5 sort of thing and every lecture is at least and hour long or coming up to an hour. Then you have three minutes before the next lecture.

S2: it does make you wonder because I’m sure they have done loads of research on this but an hour is too long isn’t it for everyone to concentrate so you think they could break it up in the middle or..

S3: there must be a reason why they have them for 50 minutes,

S2: But also thinking about memory, don’t they say you can only remember about 3 or 5 things in any lecture, can’t they say, these are the five things to remember and then if you can remember the key things then you can just fill in some detail later for the KT you can go right, this is important bit you know, these five things, and you come to the KT and you really understand what that really means and just add in details.

I: is there any way, you are saying 20 minutes and then break, is there any way that lecturers could break it up for you.

S2: They would have to police it carefully. Or maybe ask questions or do something different. Do something different, yeah that is true, a little quiz or something.

S6: we had a tutor on our access course who used to take a break after about 20 minutes and he just used to say oh we are breaking for a few minutes and would just kind of turn around and do something on the computer. And actually it’s a bit of a recharge isn’t it. Stop thinking about that for a minute, think about something else, then go back to it. And it was quite useful.

S4: RD does that sometimes, he has like a stop sign, to discuss the issues, sometimes he skips them because he is too tight for time.

S3: When I first had RD he actually used those stop signs properly.

S5: not any more.

S3: Like he gave us a couple of minutes break to do the question on the stop page and then he would ask people for their answer and that was really useful.

S1: and I found that generally the question on a stop question, one of the questions in that lecture will be in the KT. (agreement)so even if I didn’t have time to look over the whole lecture I would just look at those stop things and just look at those questions and be like okay so what’s the answer to that question, what’s the answer to that question, so you’d be like oh, I’ve got one answer for the KT.

S2: And he does really clear objectives as well. He’s been skipping over those breaks though hasn’t he in the lectures.

S4: it is because of time I think,

S2: Is it? Or is it because he is not as enthused, I don’t know. A bit of both.

S4: Maybe that, I don’t want to speculate.

S1: He is a little bit less enthusiastic than he was in my first year. We may have worn him down.

S2: That’s a shame.

S1: he is a good lecturer though in terms of he is someone else who, his lecture slides are very simple, like it’s just got what you need to know and that’s that.

S2: Yes, key points

S4: it’s the nature of what he is teaching as well though. It’s not as scientific.

S2: it’s easier to summarise do you think.

S3: the thing I think that is impressive about RD is that he is in no means my favourite lecturer but I learn more in his lectures than in quite a few other lecturers who I prefer the style of. There must be something about the way he does his lectures which works, even though I don’t necessarily like it. That’s true, and I don’t entirely know why it is.

S2: no that’s true for me because I actually did his little quiz after, so I did that and I pretty much got most of it right and I was like, well he’s obviously doing something right.

S5: Because actually if you followed the stop signs and missed out the lecture, then you go back through the lecture, you get more, that’s how I revise his lectures, I don’t read through it I go through the questions and then try and answer the questions looking through the lecture which I find it helps it stick a little bit more. And the other thing that he does that most other people don’t is out quizzes for every single one of his lectures. They’re really good, having the answers as well it’s phenomenal and I think that’s another thing to add to the learning outcome.

S6: RG does that doesn’t he, it’s quite useful.

S2: yeah he’s good actually.

S6: and wordsearches, I really love them

S2: You need something that makes you think you are learning but is a bit more fun.

I: it’s that kind of quizzing aspect,

S2: it’s about testing yourself, that’s what you need for KTs. Recall.

S3: it’s involving you on personal level, not Just as part of an audience.

S1: But as well that is something that we learnt last term isn’t though, if you test yourself you are more likely to remember than just going over something.

S3: pretty much all of things we have said are like those revision techniques which they teach you back in school, it’s being engaged, its being active with your revision and I kind of feel as though it applies to the lecture as well, if you are engaged in the lecture, if you’re active, you are going to learn more from it. In the same way would doing your revision. So maybe there is a big overlap.

I: so for all of you, with your learning it sounds more like the teaching style and how they set up their lecture rather than the method being used almost. Does that make sense?

S4: What do you mean by the method.

I: So rather than doing flipchart or CS thing Vs slides Vs flipped classroom Vs polleverywhere. As long as someone has thought out what it is and why they are doing it.

S4: For me I think the FC would be much more feasible as a way of learning, just for me to think about, have an idea about what I am learning from a lecture before hand and try and get my head around that, orientate myself, because sometimes you go into a lecture and you don’t know what is going on. So for me FC would be really useful.

I: how much of your yeargroup would engage with a FC approach.

S4: it depends how it is done, so it depends on whether there is a Khan Academy style video or 10 minutes lecture or reading, I guess it depends.

S1: I think as well like, how many would engage, I think most people would be engaged but not so many would participate if that makes sense. So the majority of people would probably end up doing the flipped classroom, everyone would end up listening and taking things in and that sort of thing but the amount of people that would actually want to contribute their ideas and their questions and that sort of thing probably isn’t as high as everyone who would be enaged.

S2: yeah, I agree.

S4: can’t you get a blended approach where you have a like a summary prereading and then have detail in the lecture. That would be useful, even just that would be helpful for me. Just to know what I am learning before I get to the lecture.

S5: the idea of Fc does rely on the fact that they lecturer does put the slides up early enough or at least a day or 2 before the lecture.

S1: It’s useful the weekend before. To be honest though, at the end of the day I think it is just about involving everyone. In terms of methods of teaching, as long as its interactive.

I: What stops you guys from interacting?

S1: Feeling stupid. Probably saying something that looks really stupid.

I: What about everyone else, because I know I have experienced where someone asks a question and even if you know the answer you just

S2: I tend to have a tick of answering too much sometimes, I feel like sometimes I have to say shut up. S6 you are quite good at answering to so I think you can expempt yourself. I think because we are mature students, its age maybe.

S1: It might be a bit silly but I always feel like I want to answer questions more when I am closer to the front.

S2: perhaps that’s what we are doing, you forget there is people behind you I think. You feel like it is a small classroom.

S3: I think maybe because you don’t have to raise your voice and it feels much like a one on one, if you are very much forward. If you can see everyone in front of you and know you are going to have to pretty much shout to be heard then

S1: No I feel like I am defintalty more likely to answer if I am closer to the front I reckon.

S2: That is probably the problem with having the flipped lecture theatre, it doesn’t feel like an interactive experience.

S1: and when people know it is going to be interactive people either tend to gravitate towards the front or towards the back. You get that middle row gap. The middle row gap. It’s an established phenomenon I think.

I: Does anyone want to add anything else before we finish off?

S4: is there anything like apps you have experienced? Because I know that BSMS had a mobile learning strategy but I think they binned it. Do you know about that at all? Other medical schools are doing really good things with apps

I: There are, the only one that they seem to keep researching seems to be MoMed. It’s a selection of books on your phone, Dr. Companion, and you get it once you are in third year. But there are so many apps, like poll everywhere can be used, there are other ones where you can have the lecture going on and then you can do something on your phone. And interacting with the lecturer and things. And there are so many of them where, we had people from BU come in who are in charge of all of this and they are trying to spread it. There is loads going on, I just don’t think that lecturers know its available or how to use it.

S2: they are probably a bit nervous about it.

S1: there is a little bit of fear with the lecturers where if they rely a lot on technology and then it doesn’t work. And the worst lectures are the ones where the technology just fails on the poor lecturer and it just sort of dies a death.

S2: I suppose you would have to get really good apps.

S3: It has to be reliable.

I: If they make it more interactive do you think it improves your learning experience in the lecture theatre.

Personally yes.

S4: I think raising hands to answer questions would be enough.

S3: It can’t just be like a one off, it can’t just be something that you do once in the lecture for half a minute and then discard because people switch on for that moment and then switch off, you need to be persistently or regularly engaging people.

I think it was too much of a faff {polleverywhere]}.

S6: I think whatever the method I think it would help my retention if it was linked to the exam, knowing that those stop questions are always going to be on the KT. Then it will help my engagement and my retention.

I: relevance?

S2: yeah it’s got to be relevant because it won’t help my retention unless I thought it was really important as well. I think it does just sort of, you go o goody. We’re doing something, we are not just sitting here. You know I think it does just help my concentration and sort of wake up a bit and not just sit there.

S6: all those hours we just sit there.

S3: So maybe like asking questions of the audience that could feasibly be on the exam, just straight up, don’t be like. This is the sort of exam question that could be on it.

S1: knowing how they could ask a question.

S3: the integration of topics as well. Intergrated questions in the exams, They touch on all the stuff that has been said by different lecturers. So you kind of get a flavour of where they are expecting you to go.

I: Do you think if there was more consistency with people asking you questions then they would get a better response from your yeargroup?

S*: Probably

S*: They probably would

S*: We’d probably get used to it.

S*: Absolutely, if it was done every lecture. People would.

S*: In a similar sort of manner as well maybe, not I’m going to shame you.

S*: You’d get used to it eventually.

S*: You might actually be surprised by what you do know if you are answering questions.

S*: Yeah, if it was in more then you probably would as opposed to every now and again you answer a question. So everynow and again the same people are going to answer because that kind of ends up being their job.

# Focus Group 2: Flipped Classroom reflections

S1: I thought the flipped classroom was great - I found it much more engaging than the standard talking at you of lectures, especially as I tend to zone out in those due to not having much of a base understanding. Having dyslexia also means that it means lectures normally go too fast for me. Having the information given before the flipped classroom meant I was able to keep on top of what was being said, not feel like I was getting left behind and therefore did not zone out. All in all, it was far more beneficial than standard lectures and id love to see more of that sort of lecture style in the future.

S2: I thought it was a valuable session in terms of teaching what might be considered more practical, clinical knowledge. I thought it would follow broadly on from the powerpoint content, but did not particularly seem to progress in this way. Quite a bit of time was spend on content that did not appear in the powerpoint presentation, although these might be considered concepts around this.

It did feel like a much more interactive session, but in terms of contributing to the acquisition of knowledge required for the KT, I'm not sure whether it achieved its aims. We are obviously so focused on grasping what we need to know for the KT and if the idea is to provide a more efficient and better means of acquiring and remembering and being able to recall this information, I'm not sure it was a complete success. I did not see a clear tie-in with a lot of what was discussed during the session and the learning objectives needed for the KT that we sat. For example, quite a lot of time was spent discussing different ways in which HRT is administered. It may of course have been the case that the particular concepts not included in the powerpoint that were discussed for quite a lot of the Flipped Classroom session could have appeared in the KT, but were not chosen this time.

I do not know what experience Dr Montgomery has had with the Flipped Classroom. She mentioned at the beginning that if we had carried out our pre-reading as we should have, the session would be very quick. As it transpired, it was a full session and she ended up doing quite a bit of the speaking - although not typical lecturn lecturing - somewhat more interactive. It didn't seem to be that this was because students had not done the required reading, more that Dr Montgomery continued to speak on, covering more content as the session went on. These topics did not seem to be necessarily guided by student input.

As a final point, as I understand the idea of the Flipped Classroom is to increase engagement and interaction by students, I feel the session did achieve this. More students than is typical were engaging vocally. I am fairly sure there were less people in the lecture theatre zoning out during the session as might happen with the usual 50 minutes of being lectured at.

S3: The flipped classroom worked well to a certain extent I felt. In the focus group we talked about how engagement was really important and this style of teaching certainly had more about it in that department. There was more dialogue than we normally expect to have in lectures and it was all the more interesting for it.

However, I don't think it was taken quite far enough. There was many more questions put to the audience than queries directed at Dr Montgomery and so to some extent it was very similar to normal lectures, just a little more fluid in style perhaps. It would have been interesting if more of the direction of the lecture had come from us students rather than the lecturer, however this most likely was due to the change in time and date of the lecture.

To get the most out of this style of lecture it seems that a substantial amount of preparatory work needs to be done by the whole audience and this is clearly not always going to be doable. Therefore I would suggest that it could only be done at times where students are not preoccupied with assessments and the like.

S4: * General comments

I'm supportive of the idea of flipped learning as a concept to further teaching methods at medical school. This is because I sometimes struggle to work out what the advantage of some lectures are compared to reading the material at home myself. So the whole idea of using flipped learning to make learning more interactive and encourage deeper understanding of ideas and concepts behind material is really appealing.

However, the success of using it at BSMS will really be down to its implementation, which will not be simple! One issue will be how best to encourage students to come to the lecture having done prior work, and whether this is done by reading or other materials such as video or audio resources. The prior work would ideally be targeted to the lecture content in some way... The other issue would be which lectures are suited to flipped learning, and I personally think it would be best used with the more difficult conceptual topics and integrating material with previous clinical knowledge that we have to enforce learning from other lectures/ modules.

* Experience with lecture

I thought the idea was good, as described above and the lecturer had clearly made an effort to think through the contents and structure of the lecture. However, I thought that because the lecturer used a flipboard and got into discussions with people near the front, a lot of the students at the back were unable to hear and see the lecture material. As such, they got a bit disengaged from the lecture which was a shame. It really reinforced that the was flipped learning is implemented has to be thought through very thoroughly for it to work.

S5: Menopause and HRT flipped classroom session

Dr. Montgomery asked students to read through the presentation slides before the lecture, late cancellation of initial lecture meant we were given two opportunities to do this and so I did. I'm not sure if this contributed to the success (in my opinion) of the session but it definitely helped me gain more from the lecture than I usually would. I rarely say much out loud in the lectures anyway but I felt confident enough during this session to contribute if asked. As for the rest of the students, I think there were definitely more people participating and answering questions then than in usual lectures.

There were no learning outcomes in the lecture slides, but there was a brief summary. The summary was of contents from second half of lecture. However, I found the lecture allowed me to see the broader picture and not get lost in the detail much in the same way knowing the learning outcomes would.

Dr. Montgomery was assisted by two flip charts and writing assistants, I think this was also vital as it allowed for the session to run without too many interruptions for writing things down, the 2 flip charts were also useful in separating information like definitions and allowing you to see the information side by side (treatment/management vs how to give HRT). I've got pictures if you would like a copy.

The session was not captured by Camtasia, and I'm not sure this would be possible as you can't always hear what members of the audience are saying. Keeping in mind the two writing assistants, there may have been at least two people that would have wanted it to be recorded for future reference.

Lecture content - all slide contents were covered during the session even though the slides were not up on the main screen. I had a print out of the slides which I was adding my notes to, I think this was very helpful for me. Additional content was also covered around 'different types of studies and their purpose', I felt that this was very useful but considering that the lecture overran, this part could have possibly been more condensed. Other additional content was also covered about pharmacodynamics (28day vs 21days & 7 day break) of HRT and contraceptive/combined pill, I felt that this was also very useful and maybe it can be added to the presentation slides for next year.

My overall feedback of the session is very positive. However, it hinges a lot on being familiar with the contents of the session as we have encountered some of it earlier in the theme but also from reading it prior to the lecture. I definitely got more out of the session than I usually do in other lectures (even if I have had the opportunity to read the lecture slides prior to the lecture which does happen but only very occasionally).

S6: I found the flipped classroom one of the best learning experiences I've had and if all of our lectures were in this format it would help from several perspectives.

It would force (visiting) lecturers to keep to a manageable number of slides because they would have to take responsibility for actually interactively teaching the information to us & would therefore have prompts from us to know which bits of info require greater investment of time - live.

It would also help because every tutor would have to ensure the material was uploaded onto Student Central in time so that we could read in advance.

I normally write notes during lectures - not necessarily to return to in revision but to help my understanding & focus during the lecture. I didn't need to do this in the flipped classroom because my attention was fully engaged throughout plus we had 2 scribes so there was even a set of take away notes to photograph.

It felt thoughtful and as if time and care had been invested in our learning & the response from students was unanimously to match this. We have many lecturers who obviously know their stuff but maybe haven't considered so well how to help us to know it too! We have had tutors who have delivered 100 slide presentations. It's a waste of everyone's time. If a 100 slide lecture was taught as a flipped classroom approach it would quickly become clear to both tutor and students that further processing by the tutor is required before the material should be presented.

I think that the tutors who enjoy the challenge of teaching and who think about how we receive and process material are the stars at med school! The subject matter is irrelevant.

So to summarise- I found the flipped classroom dynamic and interactive and ensured for me that a strong memory of information for that session was made. It felt mutually respectful because it provided and demanded back investment of attention and careful reflection and participation. I would welcome it much much more.

Possible disadvantages - as you know some of our days are packed with 7 back to back lectures. The level of intensity might be too high in which case a mixed timetable of practical sessions mixed in with lectures could help with this.

I found your module tutorial on Primary Amenorrhoea really helpful in the same way and when it came to revising for the KT it was lovely to discover that I'd retained most of the info from that session just in my memory.

# Focus group 3

I: I would like you to tell me about a lecture that you have learnt a lot from and engaged with within this module.

S4: One that was at near the start that PG gave, really in a way that you could pick up, had loads of information that wasn’t on his slide, but he paused perfectly so you got down all the information that he said that wasn’t on the slide, yet he went fast enough that you didn’t get bored. And he just got the pace completely right.

S3: Was that the first lecture or the second one?

S2: That was good.

S4: So that was the first menstrual cycle one so he was going through kind of like the menstrual cycle and how all the hormones go up and down so it was quite complex, yet his slides weren’t that detailed and you still got everything that you needed to know really easily.

S3: He just structured it really well so to start with he went through what happens to the hormones in the menstrual cycle and then explained that fully and then he took us thorugh what happens to the womb lining during the menstrual cycle and took us through the whole thing, and what happens to the cervical mucus, during the cycle, rather than. He could have done it day by day so I think it was better that way.

S6: I liked that one because on one slide he just had the picture of the graphs, which you kind of look at and you kind of freak out a little bit. Because there is just so much on it. But then by the end of it I had done a lot of writing and it was all structured so well so when I went back over it just went so smoothly.

S*: Do you want to know about another lecture or still this one?

I: No, go on, another one if you want to.

S7: CS first, what are they called, embryology lecture where she went through it all in writing with pictures but then she made us actually model it out with play dough at the end and that was really helpful.

I: Why was it helpful?

S7: Because it is quite a complex subject and you can’t really imagine it but, and I think where you have been through it already in pictures, it just sort of reinforced it at the end and you sort of remember more of what you made than what you’ve..

S6: It visualised it.

S4: I like the playdough bit but lecturers who kind of just read off the points of the slides, I don’t really find very helpful. I only found the playdough bit helpful, lecturers who just say the points. There is not really much point in me sitting here if you are not explaining it more than just the points on the slides.

S2: the majority of them are like that.

S4: CS is particularly good at trying as many different resources. And she really does try. Like some people don’t necessarily get what she is trying to do but she is always trying to think of different ways rather than just reading off the slides, you know we’ve had strawberry laces last year for the coronary arteries.

Bringing her kids in

S2: I think the problem with that is that she spends 10 minutes on that and then when you are going through it you find she is going through very complex points very quickly or skips it entirely or doesn’t define a term and I don’t know what that is, so I agree but I think that needs to be done quite quickly.

S6: I know what you mean, because when she brought her kids in it was great but I didn’t learn that much she spent a lot of time, it was really cute, and it was really cute and it was really nice seeing the kids playing around and seeing it on them but for the amount of time it took I didn’t learn nearly as much so.

S2: It’s a balance isn’t it.

S1: I think sometimes with the lecturers who just read of the slides, the issue is, if you don’t understand the slides and they literally just read of the slides then actually its then hard to go and even sometimes look it up. Because you don’t understand what they mean to go and look it up and then, if you don’t know quite how much detail to go into whereas sometimes if they kind of go a little bit off topic it kind of actually makes it, you can relate it all back. Which is something quite a lot of the lectures fail to do.

S*: I can see how JM lecture would have worked, just then, the FC one, lectures like that where if you don’t understand something and then you go through it before then you have the opportunity to ask questions and really get them to explain it but I never go through it before.

S2: That’s the issue.

S5: It’s like an all or nothing thing, if it was taught from day one, that we should be going through the lectures before the lecture and that that’s fundamental and that is vital. If we turned up to the lecture without going through it and understand it then that would be okay but you can’t pick and choose which lectures we have to go through before.

S3: I think that is too hard.

S1: I think one of the things is that you are always, it’s like going through lectures, you always get people who aren’t going to have gone through it before. And I’m not going to lie, I didn’t have a full look at it. I kind of glanced at it and saw it said menopause. But I actually found that I didn’t, I still understood it because the way she went through it, she covered the majority of it anyway so when I go back over it I think that I have got enough of the gist of it now to actually fit it all together. I’m not sure for me that is going to be just as helpful.

S4: I really like JM but I got really quite confused during that lecture, I understood the first bit, following it with the lecture that I had printed out but I’m not sure whether that is what you are meant to do or whether you are not meant to have the lecture in front of you at all.

S2: I think she could have explained it a little bit more clearly what she was doing, because I couldn’t read it either. It made my eyes hurt.

S4: the different types of oral HRT they weren’t spoke about on the slides, and that’s the bit that I personally struggled with.

S6: If she, I think it would have been better if she hadn’t given us notes and then we all would have been focusing completely on what she was doing, writing down as she went. Or give us say, really well structured, simple, almost like a template, like to fill things out on.

S2: Halfway between an MT and a lecture really. I wasn’t really clear on which way it was going.

S6: I think everyone followed the first bit really well and it got about halfway through and everyone went, no I’m lost now.

I: which part did you get lost on?

S3: If you are saying, you know the different types of oral contraceptive pills, you know when she had the graphs with the lines on, if that’s not in the lecture slides then I think I am going to really struggle to revise it, not to learn it, but to learn it for the exam now.

S2: There were visual moments when she needed a big visual, where you couldn’t see and it wasn’t that well drawn and it was like, I can’t see that.

S*: Especially for those people sitting at the sides, who were like, they couldn’t really see what was going on. It was a bit pointless.

S*: I think that approach allowed people to ask more questions, and like, people don’t normally ask questions in the lectures, but because it was more relaxed people were asking more and getting like more understanding which helped other people learn I think.

I: how much do you think you learnt from that lecture,

S3: I think for me personally, I engaged more because she was asking questions whereas in the lecture where they are just off like, going and talking I can just zone in and out. Because she was asking questions and she got people involved I actually paid more attention. And understood it better, for me personally.

S2: Did you do the reading before, if you don’t mind me asking?

S3: I didn’t, no.

S6: Just even just people asking questions sort of, because it is something different you tune in a little bit more, even if it just her repeating something, it just puts in your mind a little bit more.

S*: Like just interaction

S*: Then I agree with what she said at the start that actually she chose quite an easy lecture to that on, and if it was like a more complex topic. Say if it was like one of HW about like ions or something, ion channels and balances or something, I would not have learnt more from that method.

S2: There is a danger of going off on tangents and taking too long, which happened today. That was straightforward, people asking questions, people getting things straight but we would need 2 hours to cover that. But then if you had less lectures and we made them longer then there would be more … you can’t maybe force it into an hour.

S*: There would be more understanding.

S7: I think it is a good approach but I think there are a bit too many of us to work as like a regular thing because, so I was sat like quite far back and I couldn’t really see the board and if like people started talking or asking questions then sometimes you do just kind of zone out. It was nice as a one off I think but I don’t think that would work for that sort of group size all the time. And like the majority of people don’t read the stuff before which is our own fault but.

S3: I don’t think it would work in a MT either because we’re in the same MT group and no one says anything, people say absolutely nothing, We will sit there for like a good minute in silence before someone answers a question. I literally don’t know the answer, but I just answer the question just to say something.

S4: If I knew the answer I would definitely say but sometimes I just haven’t looked at something beforehand so it’s my own, completely my own fault, that I don’t know it. But I know that there are people in the class who know the answer, but who are too embarrassed to say it.

S3: there are people who will have always done the work. Who have read it an always know.

I: So why do you think people don’t answer?

S1: So I think, some of it is, the people, I have done the work it’s just like I don’t, not that I know most of the people haven’t its like if everybody else hasn’t done work I don’t want to be the only one who is kind of gone away and swatted up beforehand. So yeah.

S5: I would be completely opposite, if I had done the work I would come in and I would show everyone that I had done the work, It’s just I never do the work.

S*: I genuinely understand things and I know there are people who genuniley do and I am like, please, like help me, explain it to me. I wonder if it is like a social thing, isn’t it. It’s like they don’t want to look. Sometimes people don’t want to look like, it’s like the classic medical school thing of like oh I haven’t done any of my essays, I literally haven’t done any work. I’m always like, no you don’t understand, I actually haven’t, If you have then please help me. People are like oh I have no idea about what HW was talking about, okay you did but I didn’t. Please explain to me.

I: S1, you were going to say something just then.

S1: I was going to say, just going back to the point the S4 made a while ago, is that in MT, maybe in that they could have done, not almost like a prompt sheet, but like, my physics teacher always like used to like give a sheet before hand which almost had like fill in the gap type things so you kind of gave our lessons in like a lecture mode, but you had the majority of the notes there. But you did have to listen, because he wouldn’t have given you all of it. So it’s just like that prompt sheet so you’ve got like most of the work, but there is stuff that you do need to go and like fill in and I think that would have been really good, but would have also given her a bit structure, not structure because she obviously had a structure with the answers. It means we kind of know what is coming up next. Whereas then I felt like I wasn’t quite sure which direction she was going to go off at certain points. Whereas if I had kind of had. Well I had the lecture slides to look at but she wasn’t always exactly following them. So I don’t know what people think of that.

S4: That’s actually reminded me that I used to have that in my last degree, some of the lecturers, they used to print out the lectures for us, so we would pick up a handout and fill it in. And print it out and some of them would leave out blanks, or specifically not put stuff, certain bits in, so that we paid attention, and had it down and they, and maybe, I can’t remember exactly but I’m not sure if they would even put it up before the exam, it was the case, you come to the lecture and then you’ve got it. Although it is a bit cutthroat, here you go, theres a blank if you miss it that’s it. But people will pay attention if there is a blank, because actually, fundamentally, they do want the information.

S***: I think that would be a good idea.

S6: I think in like the lecture we have just had, like a template or something would be really, a lot easier to follow, filling things in so we like knew where we were. And if they had like the drawings out and we filled in details around it then that would be good.

S3: Yeah, writing stuff down makes you remember it better.

If they had the drawings with like blank labels, and you had to label it.

S6: Like literally just a line. Or something would have helped me.

S1: and I think it that one if she had put say so benefits and we so, she could have put a slides up but just have a thing that says benefits, with the bullet points then we kind of know that there is kind of how many benefits we are looking for and that kind of thing. Cos I didn’t want to almost look at the slides because I was like, I want to work it out for myself. Cos I felt like for me, I hadn’t looked at them. Because I hadn’t looked at my notes, part of it was using my brain a bit. Whos, is it the lecturer before hand who does the quiz at the end. AC has a quiz at the end and literally she has like 4, no maybe 12 questions, quite quick ones and what you do is, at the end she asks them. It’s all covered exactly in the lecture material and it’s just actually recalling that at the end. And it just kind of summarises the key points really nicely as to what she actually expects you to get out of the lecture, the important points.

S3: I do think a summary at the end does make it go over in your mind but by that time you can’t listen anymore and other students start to pack up even if you are listening you can’t listen which is really hard.

S6: Then when you are going over the questions after I generally have a quick look at them. I like it when they put like quizzes up, like I don’t do them all, all the time, but when I do, I find them really useful.

S4: Yeah maybe like summary quizzes, and a summary slide, even if they don’t go through it in the lecture because of the time that would, I know that would take a lot of work so you can’t expect every lecturer to do that but it’s good because AC are quick but really good. It’s all stuff that has just been covered. But you can do it later on if you wanted to when you do revision.

I: is there an element of what you are expecting from the session and what actually happens. What were you expecting to happen in the FC?

S1: I wasn’t expecting that.

*murmuring I had no idea/I really don’t know*

S3: I swear we’ve had something before didn’t we, when she was with the other lady.

S2: yeah we had something like that.

S3: I quite liked that one as well.

S2: she was doing the writing, not there was anything wrong with the others doing the writing apart from it was too small. But that isn’t their fault, it wasn’t that clear when she did it.

S3: I was on the back row and I could see.

S*: what makes it flipped? (15.42)

I: So the concept of the flipped classroom is, what is supposed to happen is either you are given, the slides, but what is happening in a lot of places is that you are given a 10 minute video so either JM would do it herself or she would pick one off youtube that is explained really well by someone else but has all the learning points in it. You watch it for 10 minutes so you have that background knowledge going into the lecture so once you are there it is not first contact with the information you can just consolidate what you know, ask any questions if there was any tricky topics. There is just potential for more interaction because you know what is coming up and you know what you could ask. And there is potential that she could have split you into small groups and say discuss this, here is a task on the board, do this task and then we will all come back together and do it. It is how the lecturer interprets the flipped classroom as well. The very basic thing is giving you something before hand so that you prepare. But it should be a bit more than that as well.

S4: In terms of exam, I know I shouldn’t have been thinking about it but I was. I was thinking which but, is this going to come up, is it not, because the slides and what I wrote down there was differences, which is fine you know I can learn that but in terms of I wasn’t sure whether she was going on about this oral HRT, but that wasn’t on the slide does that mean that it will come up in the exam or not come up.

I: You guys were saying you got lost at a certain point it seemed, which point was that? Why?

S1: I am just going to put it out there, I didn’t get lost at all, I think that’s because I didn’t, I was literally writing my own notes, I basically made my own mini- powerpoint presentation as I went so I was just doing like a slide for each thing, and I think that is why I didn’t get lost because I didn’t know what was on the slides so I had no idea, until now I didn’t realise that oral HRTs weren’t on the slides. So I didn’t get lost because I didn’t have any background to get lost on.

I: So I guess it was you whho were expecting and did prepare.

S*: Yeah I didn’t have the slides, I didn’t know what the slides looked like and I didn’t really get lost. Because yeah, maybe it was better not to be expecting something and not have a plan to follow.

S6: I think what you said before, if we had this sort of lecture all the time, then we kind of, I would know what to do. To work from it. But because I’m used to having the slides up there, I’m used to like, doing little notes on the slides, having things related to what is on the slides, because it wasn’t, then I got lost, I’m used to not having like the basic things on the slide.

S3: I just had all the slides up, because all the relevant headings were on the slide, I would just go and add a slide in and write down what she was saying. So I just thought it was like extra information.

S4: It’s much easier typing, you see mine are printed out. So when it got to the bit about HRT treatment you see I had no space to fit it in. And all these little diagrams,

S1: If I had printed out the slides I think I would have got more stressed by it. Not being able to. Because I have a very set way of doing my notes. And it was only because I left my notes at home this morning that I didn’t have them in front of me. So that’s organisation for you.

S3: I just thought like she would be talking about the main points that she would be wanting to get across cos its flip classroom so that would highlights to me the specific things I would need to know well and in detail in the presentation which I think is more helpful. Because there is so many slides that you can put up in a presentation, some people put up like 60 or more slides, and it is just like, ridiculous so the fact we are doing it that way she highlighted the main points, instead of just having to go through every slide. I thought that was much more helpful. Because we have learning outcomes that highlights the main points and then what she talked about just further reinforces exactly what you need to know.

S7: I don’t find the learning outcomes useful ever, I have kind of given up looking at them because I feel like they could ask you anything that they have said, that’s why I don’t find them useful.

It’s too broad

S5: the ones that are in the module handbook on student central sometimes they vary wildly from the ones that the lecturers choose, it’s as if BSMS go into the county hospital and are like right whos good at hormones. And some guy is like I’m good at hormones and they are like right, okay here you are. Will you teach them this and this. And I get that they must give the lecturers the learning objectives but like they are quite busy people they are going to teach us what they think is relevant and they have a different idea fo what should be in the lectures, compared with mr so and so who si treating really specific patients all the time. But one thing that really annoys me about lectures is, the only thing that I thought about before this session or whatever I was thinking, people, lecturers often will say have you learnt about this and we either have or haven’t, like someone said have you learnt about gut motility in this module, and I was like if you have looked it up on our curriculum, all you would need to do is look at what the 4 modules so far are that we have done and they would have seen that we have learnt about it. Or have you learnt about where the pituitary is, well yeah we did neuroscience last year, we know where it is.

S1: I don’t know, if they had just, often they will ask us so do you know about whatever and half the time people say yes, people say no, and..

S7: there is always that one person that thinks we have ,

S1: and the issue comes when some people think they know about it, and it turns out they actually don’t, some people think they don’t know about it and then they actually do and it turns out as soon as they start talk we think oh dear, we know all about this, we are now going to spend half an hour learning about where the pituitary gland is in comparison to the hypothalamus.

S3: I think people are inclined to say no because they don’t know about how much they need to know in it detail. So they are like do you know about this and it is like well how much detail do we need to know about that. It’s like oh no, I don’t know.

S1: I don’t mind people saying no, it’s just when they say yes and I don’t know. I get really annoyed about it, it’s like.

S5: It’s just the volume of people isn’t it, at least we are not in a classroom in Manchester where like there are like 400 people and one person is saying yes and 399 are like no.

S2: it’s all relative isn’t it.

S6: isn’t that a point like one person might say, oh yeah we know it or you’ll say no because we don’t know how much detail. It’s not specific questions, you can’t be like, yes I know this,

S5: I just think if they had a more basic awareness of the curriculum and the fact that like the fact that the second module is heart, lungs and blood so we probably do know quite a bit about the heart lungs and blood. If they had that then they would not be asking those questions.

S1: I think there is also a couple of instances where they could have got the same lecturer do a series of lectures, and they have got instead individual lecturers to come in and then there is massive, massive overlap. So like this time, especially with regards to thyroid, adrenal gland and a couple of the other glands there has been so much overlap, so we have talked about cushings disease, about five times, and each time they tell us something slightly different, so they will just mention two different symptoms and then when it comes up in the KT which one, who is asking this question because depending on which lecturer is asking the question I will be expected to give a different 5 set of symptoms.

S3: to be fair isn’t our module leader supposed to look at the lectures and be like, oh, maybe, do you think that someone actually and then oh these look a bit similar. Oh they’re teaching two different concepts, there should be some kind of vetting on the lectures.

S6: We’ve defintaley had this conversation to them I think, where we have been like, does anyone actually monitor what is in the lectures, how much is actually

S3: because we said that last year in the phase leaders forum to MT about JA lectures and he just said oh he is a new lecturer so we haven’t had a chance to look over them.

S6: Well that is not good enough for us.

S1: I think and just going back to if the lectures are too details or we have too many lectures on the same topic then it could be useful to have say one nice, covering one whole thing on say like the thyroid and then like actually having maybe one of these flipped where we can then go away and ask questions, and like, or just like a revision session where we can ask them all questions on it and have that for the two hours rather than two hours with two different lecturers, teach us slightly different things on the thyroid which just actually confuses us more.

S4: I do quite like the repetitiveness but some things I am like, why have they chosen this specific condition, when there are obviously loads. Is that because it is the most common, or the most interesting, but when I go through the lectures and I am like oh there is Cushings, again and again and again. That really helped me. I see what you mean about the difference, I don’t know what to do about that.

S1: it’s like, whether to have less lectures and just do it once or have it lectured over and over again and get it into our heads.

S5: I think that is a fair point, I think they think these people are teaching us different things and they are not.

S2: I think that there has been more repetition this term compared to other terms.

I: So you have had the flipped classroom today which is obviously very different to someone standing up and going through the slides, have you had anything that is not purely slide based.

S6: We had AC and JM do a kind of like that where they wrote it, they did like the menstrual cycle and they wrote that up. Which was good.

I: and how did you find that?

S6: it was good and they also gave us a sheet with a diagram on it. Which was good.

I really liked that.

S6: it was just like, to study around it and write around it, that was good.

S4: This might be really off the topic but imaging is taught in the lecture theatre with all of us and it is not really slides, it’s the interactive programme with the pointer.

I: the bright green and black interface?

S4: and I think that is the worst thing ever.

I: I am not sure how you used that one, but when I was here we had our laptops and you could click where you thought it was and then it would all come together.

It never works

We’ve had it for like two years and it never works.

S4: And you can’t get onto it yourself,

It’s a bit of a I’ll just have to hope for the best for the imaging question, it’s true.

S7: I think that’s true, that’s the one case where I would like a set of lecture slides pointing out what’s what. I don’t that interactiveness works

I: if the programme did work, do you think that kind of quizzing, and you put your answer and then they see….

I do like it, I like the idea.

S6: But you can’t take anything away, from it. You can’t revise from it.

S4: Unless you can go and do it at home.

S1: I think one of the issues with it is that we all kind of dread it cos they just have a pointer and they just hand the pointer round, so instead of us doing it on our devices and they see the groups work you are given the pointer and it is like okay so you need to, and I can’t read the question on the board because I don’t know if it is just my bad eyesight so I have to wait for somebody to tell me what I am actually pointing at and sometimes you just can’t tell what it is on the image because you have never seen it before.

S3: Like if they had a picture on the handout and they are like, oh that’s is where it is. But you don’t even have anything to write on. It’s just on the website.

S6: If they just had a slide with all the different images you could just print off the slides and circle it and annotate, it would be so much better.

S3: I know, I don’t know if this is relevant either, but at Southampton medical school with every lecture they give a handout. It’s a A4 piece of paper with a summary of everything that should be in there. With pictures and stuff and I think that would be really useful.

What keeps you engaged? What teaching methods, the way the lecturer teaches you keeps you most engaged?

S2: I was going to mention that before but I don’t know how relevant it is. But now you’ve raised it. For me it is a bit of an X Factor really, in terms of like PG lecture, I agree he’s really good, but I think he is good because he’s funny, he holds the audience, he is charismatic. A lecture who goes out there and goes bang, bang, bang, even if they are going off the slide, if there voice is monotone or bore me I can so easily with so many lecturers just zone off. The problem is, how do you teach a lecturer to do that well, I don’t know that you can so it’s like what makes a good teacher I don’t think you can. And some of them are a bit scarier than others, some people talk more in lectures you can’t hear, people start to get distracted, you start seeing facebook coming up on laptops. You start thinking oh no one else is concentrating. The lecturer probably picks up on it as well I think, Oh they’re not really following , and they’ll just race through and its just a waste of time. People start walking out sometimes. The sense of humour as well with him. If someone is quite funny and they’re really holding everyone, again it’s like I want to listen to this person I like them this is interesting. But that is a quality in a good teacher and that’s quite difficult to replicate.

S3: Do our lecturers not have teaching qualifications then?

S2: But you can’t teach that. It’s an interesting thing because I was teacher and I have taught at three different levels over the years and done three different degrees and taught very different subjects so you see people who are naturally good but presumably none of them have had teaching training I assume in terms of, they’ve maybe been taught to do medical teaching to small groups or something.

S*: Some of them are scientists or clinicians aren’t they who have got post docs, in the..

S2: But having trained as a teacher I’ve seen teachers who have had all the training in the world but I’m sure we’ve had that at school, who are rubbish because I think up to a point it’s whether you are a natural or not. Up to a point. Certainly the charisma and sense of humour, that kind of comes with personality doesn’t it.

S5: and I really think, once a lecturer comes in once holds your attention, holds the audience and then when you see them on your timetable again next time, you are like, ah yes, and if you go into a lecture thinking I am going to concentrate for that whole hour, because for me I’m really excited for RD lecture because I think he is such a good lecturer and also BF lectures this term I really liked, I am in love with her. If you go into the lecture knowing you are going to listen for the whole thing then you will. Whereas if you go into the lecture knowing that the lecture is a bit rubbishy and you don’t really, and their voice is a bit annoying and they are a bit monotone, then you are not going to concentrate.

S2: I mean HW was brilliant I always thought I’m not sure if everyone agrees but he would just stand at the front and wait for everyone to go quiet… it didn’t work for you? I think that is teaching style not him.

An example, today I thought, maybe it is because I sat closer to the front because I am getting really distracted with talking because it is the end of term. But DrAC I think she is really lovely, I think she has got a really good, I don’t know what she has got, because she is a typical example of someone standing there and just, not reading off the slides, she, I don’t know, she has a very nice manner, and everyone, unless I was just too close to the front, I thought everyone was really quiet. And I don’t know if that is because she has got some sort of authority I don’t know how you do that though.

S2: She is good, I find her though personally a little bit too fast. But that is probably me struggling, but I think she is very good.

S1: I think going back to about the different lecturers I think the thing about BF and AC is that they clearly know their stuff, and have, AC has her questions at the end and BF has her questions going all the way through and it kind of makes you listen because you know that they know, what kind of, you know they have got a goal at the end of their lecture about what they want to teach you whereas some of the lectures I feel are teaching us because they have been told we need some one to teach you this so they come in tell us all about, I know I am using the thyroid but without ever kind of reaching a goal or what they want us to learn. They’ve just kind of vaguely covered and I find that really offputting when I am listening as I don’t know what I am meant to get out of it. Where as if I am interested and know what I am meant to get out of it I am much more likely to listen and enjoy the lecture and get the stuff out of it.

S6: I think you can definitely pick up on lectures who know what they are talking about and don’t, like JA, he was sweet but..

S*: Some of them, I don’t know if they do actually want to teach us. They don’t really know how it works, they just want to get through it as quickly as possible, they’re like right, lets just do this.

S2: It’s got to be nerve-wracking hasn’t it 140 sharp people in front of I think you have got to have a certain type of temperament to be good at it and not everyone does.

I think teaching is a skill, definitely.

I: you were saying that you have lecturers who are naturally charismatic, they have got the X Factor, they can teach. But, say you have your bog standard clinician who has come in, he does a lecture, he’s probably not the best lecturer in the world. How can, what can be there to support your lecture, a bit more. Would asking questions, would quizzes, what can help the not X Factor.

S2: well that is a good point,

S3: Like a hand out that you could fill out.

*talking over each other*

I: Shall we take it in terms

S3: Yeah like they were saying earlier, like a handout to fill out. Something so I pay attention, like a quiz at the end. Just all those kid of things, to try and keep you engaged. Some kind of interactiveness,

S4: I think, the advantage that clinicians have over non-clinicians is that they have actually got real life stories, and when there are like clincians who come in and they are like this is Mr. A, he has this symptom and this symptom and teach it like that. That’s their unique selling point so they should use that to their advantage. And they don’t as much as they could.

S1: We had a really good, I was just thinking of two examples, we had one where he taught us about the ovaries, and ovarian disorders and he put up, he said there are four types of, four things that can go wrong… And he put up case by case, and the thing was we had to tell him, he put up a list of symptoms and he had to say which one of the four it was, I found that really helpful because I was listening because I knew that I was going to have to work something out, and it was a bit of a puzzle, and the other one was the lady who was talking about prescribing fertility medication, and she was, she just was just really funny and she just told it through stories, and I think that kept you engaged because a story is a story and yeah.

S7: I think just people who, it is nice to have the clinicians and that but people that use simple language, and don’t. when some people explain, these procedures and when we had a lecture on nuclear imaging, and they just talk in gibberish as if they expect us to have a really high level of understanding. Some of the best people are, even if it does seem a bit basic, you can follow. You switch off so much more quickly if you think, I’m not even understanding what they are saying, never mind what is written on the slides, so you’re just not going to listen.

Yeah and if you know the basics in the lectures, then when you go home or read a textbook , which I do sometimes then you can get a lot more out of the textbook because you can know the basics you can learn the basics properly. Whereas if they try and teach you the complicated bits and you don’t even know the basics, so when you read a textbook you are like, oh this is exactly the same. It’s like what you are introduced to first, if you are introduced to the basics first you can then learn the more complex parts but if you are introduced to the more complex parts first then you just switch off.

I: How much learning for you guys takes place in the lecture theatre?

It depends on the lecture.

S2: I think I am probably quite extreme in terms of what I get out of it, I get very little. What I find frustrating in the bigger picture is that there is lipservice paid to different types of learning methods, How we are all kinaesthetic, or this or that. I learn from reading, I don’t take much in listening first time. A little bit, maybe I am unusual like that. But I have to sit down and read it. So I have to get through the lectures, sometimes I do something else. It’s when I am in library reading through it at my own speed that. Sometimes it is just like, especially with the register, if they were not registered I am not sure how many lectures I would go to.

S6: I think what I get from lectures is the extra things they say, I think maybe I need to know that, ocassionaly I am like, oo I remember that diagram or something like that but very rarely, It’s when I go over it, like write up my notes and when I look at my notes.

S3: It really depends on the lecturer and lecture which varies massively, if they are saying helpful things around it then I am going to pay attention, and if they have got my attention then I am going to take lots of notes, and it’s really going to help me. But if they’re as I said reading it off the slides then it’s not going to help me at all.

S1: I just thought of another really good lecturer who uses interactive things and that is KC in the computer room, I know some find it really unhelpful but I find it really helpful, the way she goes around, she puts you on the spot and makes you answer a question but she does, slowly draw out the answer, and being actually in the computer room where you have got the answers and can annotate them, either on the computer or on your slides, I find more helpful that if she tried to teach it in a big lecture theatre, where I think I would turn off pretty quickly.

S2: which is true because she struggled to do it in the lecture theatre,

S*: I like, because she pretty much has a slide where she starts like introducing it, she goes round and asks a question and there is a question like every other slide and you can fill that in. when she teaches which is really helpful because you are understanding and you can write down the answer but then you can write down the explanation after it as well.

S1: Something that she does, carrying on, so she puts questions throughout. Afterwards it is meant to go up but for some reason the school office, aren’t capable. She then puts the answers up. So you do have, so it is a bit like the worksheet we were mentioning earlier, she had a bit of a worksheet like, questions to go through and you’d have the answers to look at. So you know if you have got it right or not. Cos I don’t like it when they ask questions and never tell you the answer.

S5: I think if you, we’be just slated the imaging lectures because they had around the pointer and they ask you to answer questions and all it is, is a picture on a slide and they ask you to answer a question or point it out. That’s exactly what KC does.

Its not the same, in imaging we don’t have the slides in front of us.

S1: also I think the way that KC does it is a lot less intimidating, than the way that the. Because when the imaging guy, I find the imaging guys really intimidating, when they just hand it to you and look at you, and they won’t give you any kind of hints to where it is and when you get it wrong, they’re like no.

I don’t agree I really like the imaging lecturers

I think the questions really help you consolidate your knowledge. Lots of questions throughout the lecture. I think they are the same, all of the imaging lectures.

I would say they are all absolutely completely different.

It’s hard with imaging there is nothing to take away from the imaging lectures.

With KC you do eventually get the answers.

He was really quite passive-aggressive. That is why if I got asked a question and be made to stand up in front of the screen, as in, in that little, I just don’t think I would be able to do it.

Like when he said ‘who is the cleverest person in the room’.

It was awful.

It made me feel very uncomfortable.

S2: I think going back to the flipped learning, if Julia had said today, I’ll be asking people randomly questions in the audience, now and again, just to keep you on your toes. I don’t really like it but I would have read more of it before hand. If she had gone round and kind of said, not in an aggressive way, but in a kind of, I am going to ask people, it does, you pay attention more. I think but it’s uncomfortable so. If we had that every lecture then maybe not.

S5: Well I think we are infront of, well I would like to think that everyone in the year is friends with everyone else in the year, like if I get a question wrong, like in the imaging lectures, they handed you that laser, and you got it wrong, no one is laughing at you no one is being like, ah you’re an idiot.

Because in the one with KC or whatever she would help you if couldn’t get it, she would help you with the answer, but with him, if you didn’t get it right though, or the first time, or you weren’t sure, he would just wait for you for quite a long time, I think it was a but unnecessary and a bit of a waste of time.

I: the other thing I wanted to ask you about is the use of Apps. So I used polleverywhere with you lot, I don’t know what you thought of it.

I like that.

I liked it

It’s nice as a one off,

S2: Maybe I am showing my age again, I find it a bit gimicy, really. Whatever phone I have it never seems to work and I am spending ages doing it and maybe if it came up instantly,

S*At least that way everyone on a laptop is looking at the same thing, rather than facebook or whatever, because that is the most distracting thing, composing e-mails to teachers or doing other things on their laptops.

I: What element of it? I appreciate the gimic-i-ness of it, but what element of it did you like?

S7: it gives you a bit of a break,

S*: It’s like a quiz

S*: You can talk to each other about it.

S4: And it is the sense of competeion, you know like classic medical student, who is going to get it right out of you and you friend.

S*: What was, we had a lecture or symposium, where you had to hand up a different colour, that was HW,

S*: I prefer the online thing.

S3: I was just saying I think the online thing was better because you could see what other people put as well, the percentages,

S*: I like the poll thing but I like the card thing as well, it’s a break a bit of a sort of quiz, interaction, but you are not necessarily having to say your personal answer out loud.

S*: That sort of quiz, questions but not being picked on is good.

S*: I don’t like hands up because what happens with hands up is that nobody does it.

I: I was talking to some of your colleges in one of the other sessions and they were saying that your ovarian lecture, everyone was putting their hand up, what was different about that lecture?

S7: He made us, he did it like 3 times.

S1: If they persist, it is almost if they persist, long enough and get you on side. It is all about getting us on side. Cos if we are not enjoying the lecture then we’re not going to put our hands up and we’re not going to volunteer. Whereas if we are enjoying the lecture then we are much more likely to participate.

I: And by putting your hand up and volunteering will you learn more?

S2: You might remember a bit more

S1: I think it just changes the whole kind of feel of the lecture,
